# Application of the FEM Method to Modeling and Analysis of the Cold Thread Rolling Process—Part 1: Conditions for Ensuring a Plane State of Deformations

**DOI:** 10.3390/ma16134647

**Published:** 2023-06-27

**Authors:** Krzysztof Kukiełka

**Affiliations:** Department of Production Engineering, Faculty of Mechanical Engineering, Koszalin University of Technology, Racławicka 15-17, 75-620 Koszalin, Poland; krzysztof.kukielka@tu.koszalin.pl; Tel.: +48-94-3478-478

**Keywords:** FEM, ANSYS, DEM, modeling, analysis, stresses, strains, thread rolling process

## Abstract

The article concerns the application of the FEM method for the prediction of stress and deformation states in a workpiece during the thread rolling process (TR). The analysis covered a new kinematic variant of the TR process in which the basket of the head rotates and is torque-driven, while the workpiece is stationary and the head with the rollers moves axially relative to the workpiece. The TR process was considered as a geometrical and physical non-linear initial and boundary problem with non-linear, moving, and variable in time and space boundary conditions. The boundary conditions in the contact areas of the tool with the workpiece were unknown. An updated Lagrange (UL) description was used to describe the physical phenomena at a typical incremental step. The states of strain and strain rate were described by non-linear relationships without linearization. New discrete systems of motion and deformation equations of the object in the TR were introduced, which take into account the change in the stiffness of the system during the TR process. This equation was solved by the central differences method (explicit). The material parameters were estimated during tensile tests to determine the characteristics of the C45 steel, and a new semi-empirical method was used to determine the relationship yield stress, effective true strain, and effective true strain rate in the thread rolling process. A modified Cowper–Symonds material model was also used to model the displacement process of the wedge on an elastic/visco-plastic body reflecting the TR process. A non-linear dependency of material hardening module depending on strain and strain rate was introduced. To confirm the plane state of deformation and spatial state of stress, an experimental investigation was carried out. The computer models were validated, and a good convergence of the results was obtained. Applications in the ANSYS/LS-Dyna program were developed to simulate the TR process. The developed applications enable a comprehensive time analysis of the states of displacement, strain, and stress occurring in an object consisting of a workpiece (shaft) and a tool (roller) for the case of a plane strain state and a spatial stress state. Exemplary results of numerical analyzes are presented to explain the influence of the friction coefficient on the condition of the thread quality, and the state of deformations and stresses were shown.

## 1. Introduction

The first attempts to model the process of thread rolling on a two-roller thread rolling machine with a steady rest were carried out in [1]. Formulas were developed for the required output diameter of the blank for various types of external threads (metric, trapezoidal) and the recommended bevel angles for the shaft, assuming that the volume of the material remains unchanged before and after rolling. The permissible deviations of the semi-finished product diameter were determined, depending on the required tolerance of the thread execution. Recommendations and nomograms for rolling parameters were also developed, including roll pressure force, rolling speed, and roll feed speed, as well as a selection of cooling and lubricating liquids. The formulas for forces, moments, and rolling work were given, as well as the allowable deviations of the roll profile half-angle and the allowable deviations of the roll profile pitch and tolerances of the outer diameter depending on the type of thread and its dimensions.

Olszak W. et al.’s team developed a new method of designing angular head rolls for the rolling of external threads [2]. The rollers have rolling rings with gradually increasing diameters. The rational design of these tools consists, among other things, of obtaining the desired or acceptable load conditions of individual rings, as well as of entire rollers. The dividing methods of the machining allowance between the rolling rings were developed, and the geometrical parameters of the rolls and formulas for the components of the force during the rolling of deep profiles were determined, taking into account the elastic and plastic deformation of the part material:

(1a) $$F_{y}=b_{1y}\times a+b_{2y}\times h,$$

(1b) $$F_{z}=b_{1z}\times a+b_{2z}\times h,$$

where: 

*F_y_*, *F_z_*—components (radial and circumferential) of the rolling force acting on the rolling ring,

*a*—width of the layer pressed by the ring,

*h*—hollow of the ring in the material,

*b*_1_, *b*_2_—empirical coefficients depending on other rolling conditions.

The relationship structure (1a and 1b) allowed for the determination of the forces acting on the finishing rings (when *a* = 0). The investigations were carried out using three rollers, where each of them had either one, two, or three rings [3]. Those variations allowed for different operating conditions of the outer and inner roller rings. The force acting on the outer ring is defined as half of the force acting on the roller with two rings. The inner ring was loaded with a force equal to the difference of forces acting on the rollers with three and two rings or with two and one ring. By summing up the forces acting on the active rings of the roller, the total load on the roller was obtained.

Ivanov V. [4] developed equations describing the required axial profile of rolls rolling long threads by the axial method [5,6] and gave empirical relationships for the rolling forces and moments [7,8], as well as equations of the contact surface of the part material with the active surface of the rolls in different phases of the process.

Łyczko K. [9] developed an experimental and theoretical methodology for determining the curvilinear axial profile of a tap forming a thread in (internal) holes based on the analysis of allowances measured by the volume of the material displaced through an individual root. Łyczko derived formulas for the volume of the material deformed by an individual root depending on the deformation depth. The obtained outlines were approximated by a circle, a parabola, a logarithmic curve, or by the fourth degree polynomial. Additionally, models were derived in the form of a fourth-degree polynomial for the increase in the microhardness of the thread, being formed depending on the number of roots. Łyczko derived the formulas for the recommended diameters and phases of the thread hole depending on the design and dimensions of the forming screw-tap and the type and size of the thread.

Since 2002, the application of computer modeling to the thread rolling process has been performed using the finite element method (FEM). Domblesky J. and Feng F. developed computer applications in the DEFORM-PRO v. 3.0 [10] and DEFORM-3D v. 2.3 [11] systems to simulate the rolling process of metric threads. However, the applications did not take into account the sensitivity of the material to the rate of strain and the history of the process. It was also not possible to obtain results for the complete immersion of the rolling rolls in the workpiece. This was due to the excessively large amount of non-linearities occurring during rolling and the instability of the numerical calculation process.

Pater Z. et al. applied the finite volumes method and the finite elements method in the MSC.SuperForge 2000 program for thread rolling on wood screws with two flat jaws [12], and with one flat jaw and two rollers [13].

**Figure 1:**
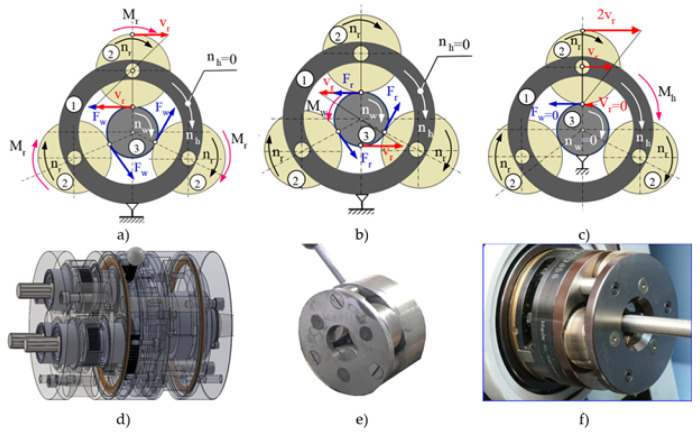
Kinematic diagrams (**a**–**c**) of three methods of rolling external threads and the rolling heads according to these methods (**d**–**f**): (**a**)—stationary basket of the head and rollers driven and a rotating non-driven workpiece that moves axially relative to the head; (**b**)—stationary head basket, rotating and non-driven rollers and rotating and driven workpiece; the head with the rollers moves axially relative to the workpiece; (**c**)—stationary object, rotating and driven basket of the head and rotating and non-powered rollers; the head with the rollers moves axially relative to the workpiece: 1—head basket, 2—rollers, 3—workpiece, $v_{r}$—rolling speed, $v_{h}$—peripheral speed of the head.

Currently, the literature describes thread rolling processes using angular heads, in which two kinematic schemes are used, as shown in Figure 1a,b.

The first kinematic variant is that the head basket (1) is stationary ($n_{h}=0$), while the rollers (2) are driven by the torque $M_{r}$ ($n_{r}>0$), which sets the workpiece (3) in rotation ($n_{w}>0$) [1,2,4,10,11,14,15,16,17] (Figure 1a). In addition, the workpiece (3) during the machining process moves axially relative to the head with the rollers. This method is used for the rolling of threads of any length and is also called the through centerless rolling method. The force $F_{w}>0$ acting on the object causes the material to move in the tangential direction, causing spatial states of displacements, strains, and stresses.

The second method is that the head basket (1) is stationary ($n_{h}=0$), while the workpiece is driven by the torque $M_{w}$ ($n_{w}>0$), which sets the rollers in rotation ($n_{r}>0$) [2,15,18] (Figure 1b). In addition, the head (1) with the rollers (2) moves axially relative to the workpiece (3) during the machining process. This method is used for rolling threads of limited length. The force $F_{r}>0$ acting on the rollers causes displacement of the object material in the tangential direction, causing a spatial state of displacements and strains.

In order to create plane states of displacements and strain in the workpiece, as well as a spatial state of stresses, the Author used an innovative method of thread rolling (Figure 1c) [15,16,17,19]. This method is based on the fact that the head basket (1) is driven by the torque $M_{h}$ and rotates at a speed of $n_{h}>0$, while the object (3) is stationary ($n_{w}=0$). The rollers (2) are not driven but rotate at speed $n_{r}>0$. In addition, the head (1) with the rollers (2) during the machining process moves axially relative to the workpiece (3). A characteristic feature of this kinematic variant is that tangential forces do not act on the workpiece ($F_{w}=0$ and $F_{r}=0$), but only normal forces in planes parallel to the axial plane act. Thus, with such a system of forces, there is no displacement of the material in the tangential direction. This method is used to rolling threads with increased accuracy but limited lengths.

**Figure 2:**
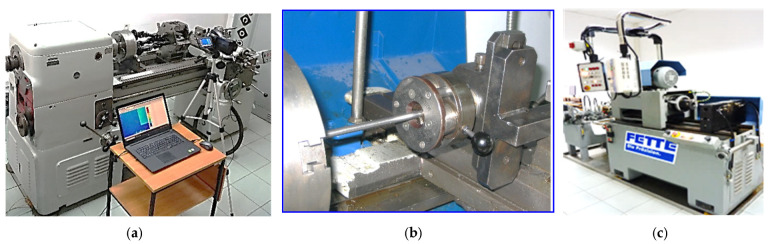
View of test stands for rolling external threads, equipped with angular heads according to the first (**a**) and second (**b**) and third (**c**) kinematic variations.

All three kinematic variants of thread rolling are implemented in the Center of New Technologies at the Faculty of Mechanical Engineering of the Koszalin University of Technology. The rolling station according to the first method is shown in the Figure 2a, and according to the second method—in the Figure 2b and according to the third method—in the Figure 2c. 

**Figure 3:**
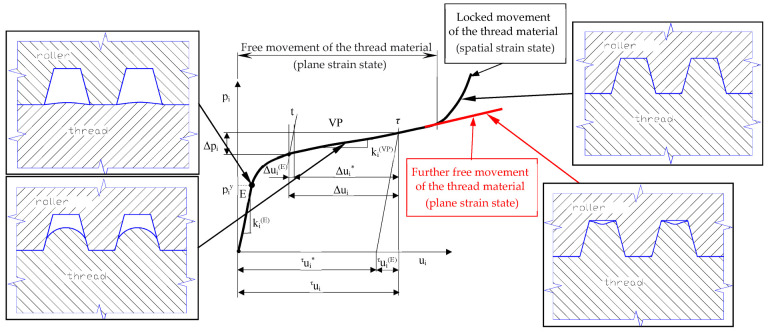
Diagram p_i_-u_i_ for the contact of the shaped thread with roller, where Δu_i_*—is the sum of visco-plastic increments, ^τ^u_i_*—components of the node’s displacement in time.

In addition, in the third method, in order to ensure plane states of displacements and strains, it is necessary to maintain the tip clearance between the rollers and the shaped thread outline shown in Figure 3. Ensuring plane states of displacements and strain of the material during thread rolling makes it possible to shape the thread outline with greater dimensional accuracy and to obtain a thread of unprecedented quality.

Other factors that adversely affect the quality of the thread are caused by insufficient accuracy of modeling of the rolling process. Thus far, the rolling process has been described by equations of motion that do not take into account the dynamics of the process or do not take into account the variable stiffness and variable damping of the system during TR process. Therefore, it is assumed that the stiffness matrix and the damping matrix are constant (K=const and C=const) [10,11,14,20].

The strain states of the part material in the TR process are most often described by conventional (relative) strains calculated from the relationship $\varepsilon =\Delta L/L_{0}$, where ΔL is the length increment and $L_{0}$ is the initial length [1,21,22,23,24,25,26,27]. However, for large deformations, the true (logarithmic) strain φ=ln(1+ε) should be used [15,16,17,18,28,29], which means that at deformations above ε=0.1[–], the differences are significant, for example, for limit values, the conventional deformation is ε=2.0[–] and the true strain is only $\varphi =ln\left(1+2\right)=1.1[–]$.

In addition, the mechanical properties of materials based on the uniaxial tensile test of cylindrical samples on a testing machine are described in accordance with the standard on a global scale without taking into account the deformation states in the neck [27], which means that the values of true plastic deformations are underestimated by up to three times. This is due to the fact that it is impossible to determine the elongation value of the sample at which a separation crack occurs inside the neck, and it is impossible to determine the actual values of spatial displacements of the material inside the neck, which are then used to calculate the true values of material strain.

The adoption of inappropriate measures of physical quantities and inappropriate methodologies for calculating deformation and stress states causes that the mechanical properties of materials are described inadequately to the requirements. The semi-empirical method proposed by the Author does not have these disadvantages. For example, for C45 steel, the true limit deformation (the so-called failure strain) calculated according to the standard is $\varphi_{f}=0.51\left[–\right](\varepsilon_{f}=0.66[–]$), while the true failure strain calculated taking into account the true ones in the neck, according to the proposed methodology, is $\varphi_{f}=1.52\left[–\right](\varepsilon_{f}=2.88[–]$).

Geometric and physical non-linearity of the system is also omitted by using inadequate material models. The use of Johnson–Cook [30] or Cowper–Symonds [14] constitutive equations means that the variability of the material-hardening modulus during machining is not taken into account and it is treated as a body with linear hardening, the so-called bilinear hardening material model. In addition, the invariability of the plastic strain rate of the material is assumed, assuming a constant value of the effective strain rate in the rolling process. Meanwhile, in the thread rolling process, the maximum effective strain rate occurs in the initial phase of contact of the rollers with the workpiece and amounts to ${\dot{\varphi }}_{e}=10^{4}\;s^{-1}$, and then decreases to ${\dot{\varphi }}_{e}=0\;s^{-1}$ in the axial plane. The real impact of the strain rate on the hardening of the material is large and increases the yield stresses by up to 20%. However, in the assumed material models, the efficiency of the strain rate is logarithmized, so, for example, for ${\dot{\varphi }}_{e}=1\;s^{-1}$, $ln({\dot{\varphi }}_{e})=0$, while for ${\dot{\varphi }}_{e}=10^{4}\;s^{-1}$, $ln({\dot{\varphi }}_{e})=9.21$, and for ${\dot{\varphi }}_{e}=10^{5}\;s^{-1}$, $ln({\dot{\varphi }}_{e})=11.51$. As a result, the calculated sensitivity of the material to strain rate is underestimated. It is also not possible to calculate the function $K_{\dot{\varphi }}$, which depends on the effective strain rate, for ${\dot{\varphi }}_{e}=0\;s^{-1}$.

In our own works [15,16,17,18,19,28,29], the incremental updated Lagrange description, variational formulation, dynamic theory of metal materials with velocity-type memory, contact mechanics, non-linear theory of continuous medium, and the Finite Element Method are used.

Our own research shows [15,16,17,18,19,28,29] that in typical thread rolling tools, when the profile is completely filled, the deformed material flows not only in planes parallel to the axial plane, but also in the tangential direction (perpendicular to the axial plane), and then there are spatial states of displacements, strains, and stresses (Figure 3).

**Figure 4:**
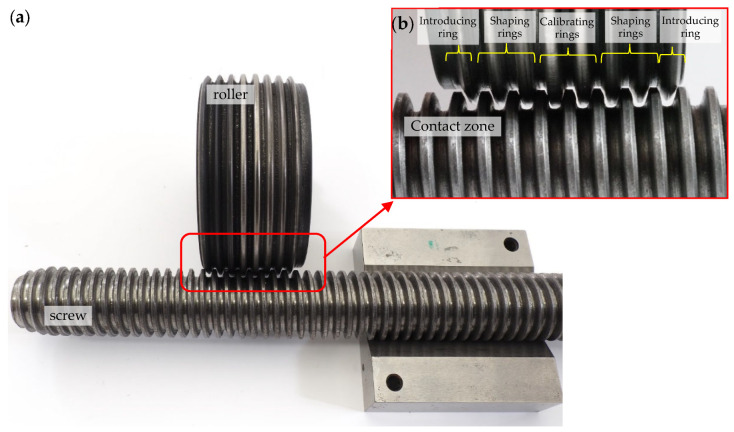
(**a**) View of a symmetrical roller with three shaping rings and two calibrating rings for trapezoidal thread Tr22×4 and a screw with a shaped thread. (**b**) View of the contact zone between the tool and the thread.

In accordance with our work, it is shown by the outline of the rolling of the thread roll that when increasing the height of the working ring, the tip clearance ensures that the top of the thread profile does not make contact with the roller. Then, with some approximation, it can be assumed that plane states of displacements and strains, as well as a spatial state of the stresses, appear in the deformed material, and there is no unfavorable jamming of the shaft material during rolling. In order to test the hypothesis, model tests, numerical simulations using the FEM method, and experimental verification were carried out (Figure 4).

Determining the states of displacements and deformations of the material in the thread-rolling process is a fundamental issue because it determines the possible simplifications not only of the numerical model but also of the model used for model investigations. In addition, reducing the complexity of the model reduces the time-consuming calculations and the demand for the computing power of computers, which is of significant practical importance when designing the rolling process. Knowledge of the mechanism of plastic material flow during thread rolling, and mainly of the states of displacement of the workpiece in various phases of the process and after unloading—the so-called spring back—are necessary for the correct design of the outline of the active surface of the kneading rolls.

The article deals with the determination of thread rolling processing conditions to ensure flat states of displacement and deformation of the workpiece material. To predict stress and strain states and the displacement and spring back of the workpiece material during and after the thread rolling process, the FEM method was used. The updated Lagrange description was used to describe the physical phenomena at a typical incremental step. Non-linear incremental models without any linearization were used to describe physical phenomena. The results of the numerical simulation were experimentally verified on the example of selected parameters of the thread.

Figure 4 shows an exemplary roller for rolling of a trapezoidal thread Tr22×4, whose geometry of the active (working) rings was modified so that during the rolling process, plane states of material displacement and deformation occur in planes parallel to the axial plane of the formed thread. 

## 2. Materials and Methods

### 2.1. Modeling of Thread Rolling Process

The thread rolling process was considered as a geometrically and physically non-linear boundary-initial problem, in which there are non-linear, moving and variable in time and space boundary conditions. The boundary conditions in the tool—workpiece contact areas are unknown.

Mathematical description of non-linear phenomena requires the use of other than in linear problems, principles of formulating boundary-initial problems and more complex methods of solving them.

Geometric non-linearity results from the non-linear relationship between strain and displacement. It is caused by a change in the geometry of the workpiece during the machining process.

Physical (material) non-linearity is caused by non-linear mechanical properties of the workpiece material, leading to a non-linear stress–strain relationship.

**Figure 5:**
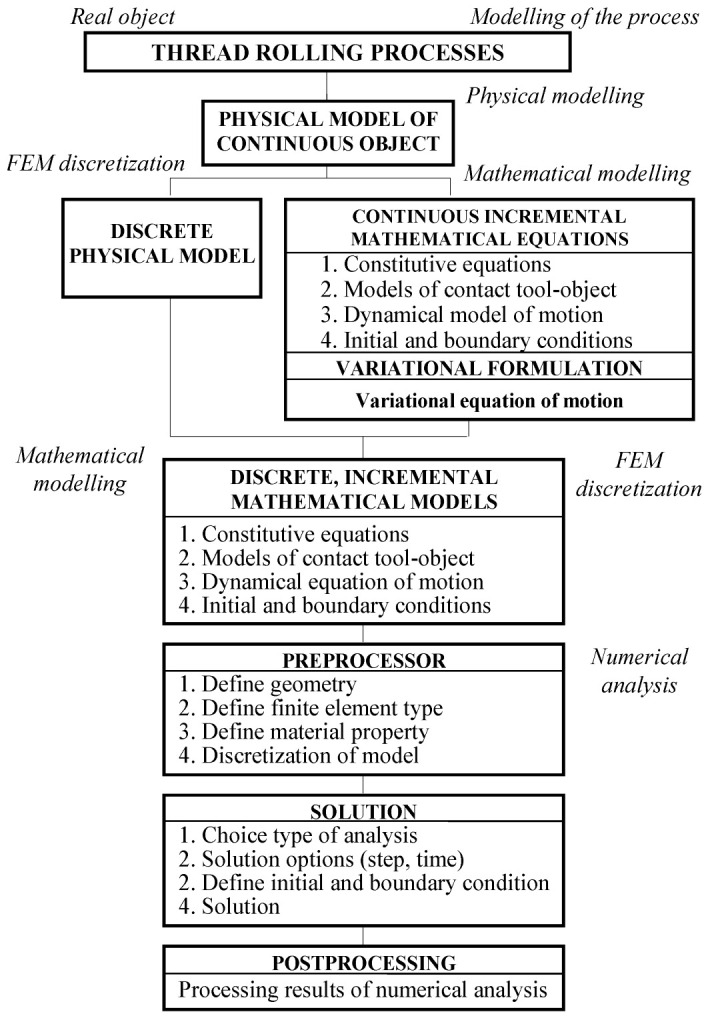
Scheme of modern modelling and analysis of thread rolling process.

For the modeling and analysis of the thread rolling process as a real object, which consists of a three-rollers angle head and a workpiece (see Section 2.5.6), a modern algorithm was used, with variational method and FEMs (Figure 5) [20,31,32,33,34,35].

### 2.2. The Concept of Incremental Description

**Figure 6:**
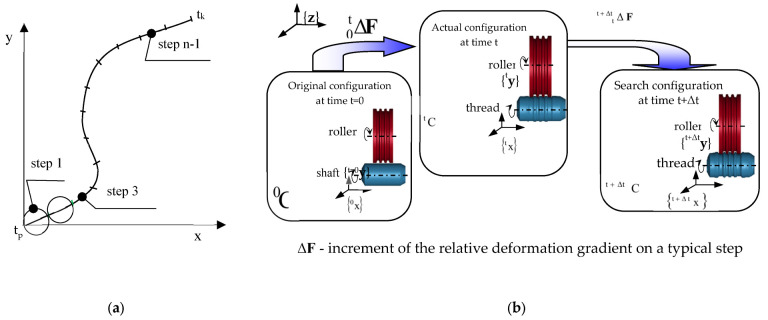
Schematic diagram of the step-by-step analysis of the thread rolling process (**a**) and the configurations adopted by the body in subsequent steps in the updated Lagrange description (**b**).

Due to the strong geometrical and physical nonlinearities occurring in the thread rolling process, the updated Lagrange description [31,32,33,34,35] (Figure 6), known in non-linear mechanics, was used. Using this description, for a typical time step τ=t+Δt, where Δt is a small increment of time, calculated on the basis of the natural frequency of the system, it is assumed that solutions for time steps in the interval [0, t] are known, and the solutions for the next moments are sought. As a result, for successive discrete moments of time τ=0,Δt,2Δt,… the geometry of the body and the states of incremental displacements, displacement velocities, accelerations, strains, strain rates, and stresses are determined.

The concept of incremental description has already been verified by the author and was used to describe other plastic-forming processes: cutting [36,37,38,39,40,41,42], thread rolling [15,16,17,28,29], burnishing rolling [43,44,45,46,47,48,49], drawing [50,51,52], grinding with a single abrasive grain [53], calculating the fatigue strength of products [54,55,56], and diamond sliding burnishing [56].

It is assumed that at the moment t, the initial (original) configuration of the body C0 and the current configuration ${\;}^{t}C$ are known. On the other hand, the dynamic equilibrium configuration Ct+Δt at the next moment τ = t + Δt is sought (Figure 6).

The application of the updated Lagrange description also requires the adoption of appropriate coordinate systems, the definition of adequate measures of increments of displacements, strains, and stresses, as well as procedures for their accumulation at the incremental step. Three systems of orthogonal Cartesian coordinates have been adopted in this paper: {x} movable object-related, {y} movable tool-related, and {z} fixed reference system (Figure 6).

### 2.3. Measures of Increments of Physical Quantities

The tool was treated as a rigid or elastic body, and the object as a body in which elastic deformations (in the range of the reversible zone) and viscous and plastic (in the range of the irreversible zone) may occur, assuming mixed (kinematic and isotropic) non-linear strain hardening. For the build a material model, abbreviated as E/VP, the non-linear Huber–Mises–Hencky (HMH) plasticity condition, plastic potential, and flow law associated with the yield surface was used. The initial states of displacements, stresses, and strains after previous operations were also introduced.

The strain state of the workpiece is described by the symmetric Green–Lagrange strain tensor Tεtt, while the strain rate state is described by the Green–Lagrange strain rate tensor Tε˙tt [24,49,50]. On the other hand, the increments of these measures on a typical incremental step are described by the increment of strain tensor ΔTεtτ and the increment of strain rate tensor ΔTε˙tτ, respectively. The increment of the components Δεijtτ of the tensor ΔTεtτ was derived from the definition of the increment of any function [56,57], and consists of a linear Δε¯ijtτ and non-linear Δε~ijtτ part, according to the Equation:

(2) Δεijtτ=Δε¯ijtτ+Δε~ijtτ=12Δui,jtτ+Δuj,itτ+12·Δuk,itτ·Δuk,jtτ,

where Δui,jtτ=∂(Δuitτ)/∂(xit) denotes the partial derivative.

In a similar manner, the following formula for the increments of the components Δε˙ijtτ of the strain rate incremental tensor ΔTε˙tτ are obtained [49,50]:

(3) Δε˙ijtτ=Δε¯˙ijtτ+Δε~˙ijtτ=12Δu·i,jtτ+Δu·j,itτ+12·Δu·k,itτ·Δu·k,jtτ

The introduction of non-linear terms means that exact formulas will be used, without any linearization.

The stress state and stress increment were described by the second (symmetric) Pioli–Kirchhoff stress tensor Tσtt and its increment ΔTσtτ, respectively.

The components $\Delta \sigma_{ij}$ of the stress increase tensor ΔTσtτ for the body E/VP were calculated from the following formula:

(4) $$\Delta \sigma_{ij}=C_{ijkl}^{\left(E\right)}\cdot \left(\Delta \varphi_{kl}-\psi \cdot \frac{A_{kl}}{B}\right)=C_{ijkl}^{\left(E\right)}\cdot \left(\Delta \varphi_{kl}-\psi \cdot \frac{{\tilde{S}}_{kl}\left\{{\tilde{S}}_{ij}\cdot C_{ijkl}^{\left(E\right)}\cdot \Delta \varphi_{kl}-{\frac{2}{3}\cdot \sigma }_{Y}\left(\varphi_{e}^{\left(VP\right)},{\dot{\varphi }}_{e}^{\left(VP\right)}\right)\cdot {\dot{E}}_{T}^{\left(VP\right)}\cdot \Delta {\dot{\varphi }}_{e}^{\left(VP\right)}\right\}}{{{\tilde{S}}_{ij}\cdot C_{ijkl}^{\left(E\right)}\cdot \tilde{S}}_{kl}+\frac{2}{3}\cdot \sigma_{Y}^{2}\left(\varphi_{e}^{\left(VP\right)},{\dot{\varphi }}_{e}^{\left(VP\right)}\right)\cdot \langle {\tilde{C}+\frac{2}{3}\cdot E}_{T}^{\left(VP\right)}\rangle }\right),$$

where ψ is the load factor and is ψ=1 for loading and neutral and ψ=0 for unloading processes, $A_{kl}$ are the tensor components and B is a scalar (hardening function), and for mixed hardening were calculated according to the following Equations:

(5) $$A_{kl}={\tilde{S}}_{kl}\left\{{\tilde{S}}_{ij}\cdot C_{ijkl}^{\left(E\right)}\cdot \Delta \varphi_{kl}-{\frac{2}{3}\cdot \sigma }_{Y}(\varphi_{e}^{\left(VP\right)},{\dot{\varphi }}_{e}^{\left(VP\right)})\cdot {\dot{E}}_{T}^{\left(VP\right)}\cdot \Delta {\dot{\varphi }}_{e}^{\left(VP\right)}\right\},$$

(6) $$B={{\tilde{S}}_{ij}\cdot C_{ijkl}^{\left(E\right)}\cdot \tilde{S}}_{kl}+\frac{2}{3}\cdot \sigma_{Y}^{2}\left(\varphi_{e}^{\left(VP\right)},{\dot{\varphi }}_{e}^{\left(VP\right)}\right)\cdot \langle {\tilde{C}+\frac{2}{3}\cdot E}_{T}^{\left(VP\right)}\rangle ,$$

In the case of kinematic hardening, formula (16) takes the following form:

(7) $$B={{\tilde{S}}_{ij}\cdot C_{ijkl}^{\left(E\right)}\cdot \tilde{S}}_{kl}+\frac{2}{3}\cdot \sigma_{Y}^{2}\left(\varphi_{e}^{\left(VP\right)},{\dot{\varphi }}_{e}^{\left(VP\right)}\right)\cdot \tilde{C}\left(\varphi_{e}^{\left(VP\right)},{\dot{\varphi }}_{e}^{\left(VP\right)}\right),$$

while for isotropic hardening—the following form is taken:

(8) $$B={S_{ij}\cdot C_{ijkl}^{\left(E\right)}\cdot S}_{kl}+\frac{4}{9}\cdot \sigma_{Y}^{2}\left(\varphi_{e}^{\left(VP\right)},{\dot{\varphi }}_{e}^{\left(VP\right)}\right)\cdot {\dot{E}}_{T}^{\left(VP\right)}.$$

In Equations (4)–(8), $\Delta \varphi_{kl}$ are the components of the increment of the tensor of total true strain (elastic, plastic, and viscous), $C_{ijkl}^{\left(E\right)}$ is the fourth-order tensor characterizing the elastic properties of the object material, ${\tilde{S}}_{ij}$ is the cumulative component of the stress deviator in the time considered t, $E_{T}^{\left(VP\right)}$ is the strain-hardening modulus at the beginning of the step, ${\dot{E}}_{T}^{\left(VP\right)}$ is the strain rate-hardening modulus at time t, $\tilde{C}\left(\varphi_{e}^{\left(VP\right)},{\dot{\varphi }}_{e}^{\left(VP\right)}\right)$ is the temporary modulus of translational hardening, $\sigma_{Y}(\varphi_{e}^{\left(VP\right)},{\dot{\varphi }}_{e}^{\left(VP\right)})$ is the temporary yield stress of the object material, depending on the temporary values of the effective visco-plastic true strain $\varphi_{e}^{\left(VP\right)}$ and visco-plastic true strain rate ${\dot{\varphi }}_{e}^{\left(VP\right)}$.

The yield true stress model $\sigma_{Y}(\varphi_{e}^{\left(VP\right)},{\dot{\varphi }}_{e}^{\left(VP\right)})$ takes the following form:

(9) $$\sigma_{Y}\left(\varphi_{e}^{\left(VP\right)},{\dot{\varphi }}_{e}^{\left(VP\right)}\right)={K\cdot (K}_{\varphi }{)}^{n}{\cdot (K}_{\dot{\varphi }}{)}^{n_{1}},$$

where $K_{\varphi }$ and $K_{\dot{\varphi }}$ are one-parameter functions of yield stresses depending on strains and strain rates, respectively, with the boundary condition that for ${\dot{\varphi }}_{e}^{\left(VP\right)}=0,$ $K_{\dot{\varphi }}=1,$ must be met. The functions $K_{\varphi }$ and $K_{\dot{\varphi }}$, in the general case, have the following forms:

(10) $$K_{\varphi }=\varphi_{0}+{{a\cdot (\varphi }_{e}^{\left(VP\right)}{)}^{3}+{b\cdot (\varphi }_{e}^{\left(VP\right)}{)}^{2}+{c\cdot \varphi }_{e}^{\left(VP\right)}+d\cdot (\varphi }_{e}^{\left(VP\right)}{)}^{n_{2}},$$

(11) $$K_{\dot{\varphi }}=1+e\cdot {\left({\dot{\varphi }}_{e}^{\left(VP\right)}\right)}^{3}+f\cdot {\left({\dot{\varphi }}_{e}^{\left(VP\right)}\right)}^{2}+g\cdot {\dot{\varphi }}_{e}^{\left(VP\right)}+h\cdot {(\dot{\varphi }}_{e}^{\left(VP\right)}{)}^{n_{3}}.$$

The material constants $a-h,n_{1}-n_{4},K\;\mathrm{and}\;\varphi_{0}$ in the yield stress models (9), (10), and (11) were determined in experimental studies, and for C45 steel, are listed in Section 2.5.4.

The increment of the yield stress at a typical step t→t+∆t is defined by the following equation [44]:

(12) $$\Delta \sigma_{Y}\left(\varphi_{e}^{\left(VP\right)},{\dot{\varphi }}_{e}^{\left(VP\right)}\right)={\left.\frac{\partial \sigma_{Y}\left(\varphi_{e}^{\left(VP\right)},{\dot{\varphi }}_{e}^{\left(VP\right)}\right)}{\partial \varphi_{e}^{\left(VP\right)}}\right|}_{{\dot{\varphi }}_{e}^{\left(VP\right)}=const.}\cdot \Delta \varphi_{e}^{\left(VP\right)}+{\left.\frac{\partial \sigma_{Y}\left(\varphi_{e}^{\left(VP\right)},{\dot{\varphi }}_{e}^{\left(VP\right)}\right)}{\partial {\dot{\varphi }}_{e}^{\left(VP\right)}}\right|}_{\varphi_{e}^{\left(VP\right)}=const.}\cdot \Delta {\dot{\varphi }}_{e}^{\left(VP\right)},$$

where $\Delta \varphi_{e}^{\left(VP\right)}$ and $\Delta {\dot{\varphi }}_{e}^{\left(VP\right)}$ are the increments of the visco-plastic effective true strain and the effective true strain rate, respectively.

**Figure 7:**
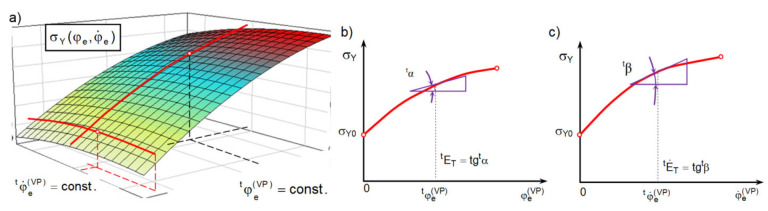
The graph of relationship $\sigma_{Y}\left(\varphi_{e}^{\left(VP\right)},{\dot{\varphi }}_{e}^{\left(VP\right)}\right)$ (**a**), and one parametric curve $\sigma_{Y}\left(\varphi_{e}^{\left(VP\right)}\right)$, for φ˙teVP=const (**b**) and definition of the partial derivative $[\partial \sigma_{Y}{(\varphi }_{e}^{\left(VP\right)},{\dot{\varphi }}_{e}^{\left(VP\right)})/\partial \varphi_{e}^{\left(VP\right)}]≅tg\alpha$ and one parametric curve $\sigma_{Y}\left(\varphi_{e}^{\left(VP\right)},{\dot{\varphi }}_{e}^{\left(VP\right)}\right)$ for $\varphi_{e}^{\left(VP\right)}=const.$ definition of the partial derivative $[\partial \sigma_{Y}{(\varphi }_{e}^{\left(VP\right)},{\dot{\varphi }}_{e}^{\left(VP\right)})/\partial {\dot{\varphi }}_{e}^{\left(VP\right)}]≅tg\beta$ (**c**).

In order to further evaluate the above expression for $\Delta \sigma_{Y}\left(\varphi_{e}^{\left(VP\right)},{\dot{\varphi }}_{e}^{\left(VP\right)}\right)$ from (12), it is necessary to obtain partial derivatives $\partial \sigma_{Y}\left(\varphi_{e}^{\left(VP\right)},{\dot{\varphi }}_{e}^{\left(VP\right)}\right)/\partial \varphi_{e}^{\left(VP\right)}$ and $\partial \sigma_{Y}\left(\varphi_{e}^{\left(VP\right)},{\dot{\varphi }}_{e}^{\left(VP\right)}\right)/\partial {\dot{\varphi }}_{e}^{\left(VP\right)}$ from the data obtained in a series of tensile tests at different true strain rates using virgin material specimens (see Section 2.5.1). These partial derivatives are calculated using a geometric interpretation of the derivative [44]. Then, the partial derivative at a given time t is equal to the tangent of the angle of inclination of the tangent to the corresponding hardening curve at time t: $\left[\frac{\partial \sigma_{Y}{(\varphi }_{e}^{\left(VP\right)},{\dot{\varphi }}_{e}^{\left(VP\right)})}{\partial \varphi_{e}^{\left(VP\right)}}\right]≅tg\alpha ,$ for ${\dot{\varphi }}_{e}^{\left(VP\right)}=const.$ and $\left[\frac{\partial \sigma_{Y}{(\varphi }_{e}^{\left(VP\right)},{\dot{\varphi }}_{e}^{\left(VP\right)})}{\partial {\dot{\varphi }}_{e}^{\left(VP\right)}}\right]≅tg\beta$, for $\varphi_{e}^{\left(VP\right)}=const.$ (Figure 7).

### 2.4. The Equation of the Motion of a Discrete Object on a Typical Step Time

The variational, non-linear equation of motion and deformation of the object was derived using the stationarity condition of the functional, which is the incremental of the total energy of the system, at a typical step time. This equation has an entangled form, and in order to solve it, a spatial approximation appropriate to the finite element method was used, obtaining discrete systems of an equation of motion and the deformation of a typical finite element and for a typical time step t→τ=t+Δt in the thread rolling process. By writing the specified equations of motion for all finite elements separated from the tool and workpiece after their transformation to a common global coordinate system {z} and after expanding and summing over all finite elements, the equation of motion of a discrete object in the updated Lagrangian description in a typical step incremental was obtained with the following form [44]:

(13) $$M\cdot \Delta \ddot{r}+C\cdot \Delta \dot{r}+\left(K+\Delta K\right)\cdot \Delta r=\Delta R+\Delta F+F+R,$$

where: M—temporary mass matrix; C—temporary damping matrix; K, ΔK—respectively, temporary stiffness matrix and its increment; F, ΔF—respectively, temporary column vector of the internal loads of nodes and its increment; R, ΔR—respectively, temporary column vector surface loads and its increment; Δr—column vector of node displacement increments; $\Delta \dot{r}$—column vector of node velocity increment; $\Delta \ddot{r}$—column vector of node acceleration increment.

The System of Equation (13) contains N equations differential of the second order with constant (incremental step) coefficients in which 2N known elements of the vector of internal forces F and external forces R and $3N^{2}$ elements of the matrix M, C and K occur, but it contain $s4N+N^{2}$ unknowns, i.e., the components of the vectors: an increment of displacements of nodes Δr, an increment of velocity of nodes $\Delta \dot{r}$, an increment of node accelerations $\Delta \ddot{r}$**,** an increment of the internal loads of the object ΔF, and $N^{2}$ unknown elements of the object stiffness increment matrix ΔK. In this equation, it is also a part of the components of the vector of increase of external loads ΔR in contact areas that is unknown.

Equation (13) was solved using partial linearization and the method of central differences (explicit), in which the differential approximation of the derivatives of partial displacements is assumed according to the following Equations [32]:

(14) $${\dot{r}}^{t}=a_{1}\cdot \left(r^{t+∆t}-r^{t-∆t}\right),$$

(15) $${\ddot{r}}^{t}=a_{2}\cdot \left(r^{t+∆t}-{2\cdot r^{t}+r}^{t-∆t}\right),$$

where $a_{1}=\frac{1}{2\cdot ∆t}$ and $a_{2}=\frac{1}{∆t^{2}}$ are the integration constants, then Equation (13) takes the following form:

(16) $$\tilde{M}\cdot r^{t+∆t}=\tilde{Q},$$

where $\tilde{M}$ is the effective mass matrix and $\tilde{Q}$ the effective load vector:

(17) $$\tilde{M}=a_{2}\cdot M+a_{1}\cdot C,$$

(18) $$\tilde{Q}=R+F+2\cdot a_{2}\cdot R\cdot r^{t}-\left(a_{2}\cdot M+a_{1}\cdot C\right)\cdot r^{t-∆t}.$$

From Equation (15), the unknown column vector of the displacements of the nodes of the discrete object at the end of the considered time step $r^{t+∆t}$ is calculated for given initial $\left\{r\left(t=0\right)\}=\{r_{0}\right\}$, $\left\{\dot{r}\left(t=0\right)\}=\{{\dot{r}}_{0}\right\}$ and boundary values for the case of the thread rolling process.

In the already formulated constitutive relationships (material models) Equations (2)–(8), the Cauchy index notation was used to facilitate mathematical operations. In order to use these relationships for the Object Motion Equation (16) derived and to develop computer applications for a numerical simulation of the thread rolling process, it is convenient to write them in a matrix form.

Relationship (4) now takes a matrix form. The increment of the stress tensor $\Delta T_{\sigma }$, for mixed hardening, is calculated from the following formula:

(19) $$\Delta T_{\sigma }=C^{\left(E\right)}\cdot \left(\Delta \varphi -\psi \cdot A/B\right)=C^{\left(E\right)}\cdot \left(\Delta \varphi -\psi \cdot \frac{\tilde{S}\cdot \left\{\tilde{S}\cdot C^{\left(E\right)}\cdot \Delta \varphi -{\frac{2}{3}\cdot \sigma }_{Y}{(\varphi }_{e}^{\left(VP\right)},{\dot{\varphi }}_{e}^{\left(VP\right)})\cdot {\dot{E}}_{T}^{\left(VP\right)}\cdot \Delta {\dot{\varphi }}_{e}^{\left(VP\right)}\right\}}{\tilde{S}\cdot C^{\left(E\right)}\cdot \tilde{S}+\frac{2}{3}\cdot {[\sigma }_{Y}{(\varphi }_{e}^{\left(VP\right)},{\dot{\varphi }}_{e}^{\left(VP\right)}){]}^{2}\cdot \langle {\tilde{C}}_{T}^{\left(VP\right)}\varphi_{e}^{\left(VP\right)},{\dot{\varphi }}_{e}^{\left(VP\right)})+\frac{2}{3}\cdot E_{T}^{\left(VP\right)}\rangle }\right),$$

where $C^{\left(E\right)}$ is the Hook matrix, Δφ is the column vector of Green–Lagrange of total true strain increment, **A** is the matrix, and B is the scalar, according to the formulas:

(20) $$A=\tilde{S}\cdot \left\{\tilde{S}\cdot C^{\left(E\right)}\cdot \Delta \varphi -{\frac{2}{3}\cdot \sigma }_{Y}{(\varphi }_{e}^{\left(VP\right)},{\dot{\varphi }}_{e}^{\left(VP\right)})\cdot {\dot{E}}_{T}^{\left(VP\right)}\cdot \Delta {\dot{\varphi }}_{e}^{\left(VP\right)}\right\},$$

(21) $$B=\tilde{S}\cdot C^{\left(E\right)}\cdot \tilde{S}+\frac{2}{3}\cdot {[\sigma }_{Y}{(\varphi }_{e}^{\left(VP\right)},{\dot{\varphi }}_{e}^{\left(VP\right)}){]}^{2}\cdot \langle {\tilde{C}}_{T}^{\left(VP\right)}\varphi_{e}^{\left(VP\right)},{\dot{\varphi }}_{e}^{\left(VP\right)})+\frac{2}{3}\cdot E_{T}^{\left(VP\right)}\rangle .$$

The increment of the total strain tensor $\Delta T_{\varepsilon }$ is calculated from the following formula:

(22) $$\Delta T_{\varepsilon }=\frac{1}{1-{\tilde{S}}^{**}}\cdot {(D}^{\left(E\right)}\cdot \Delta \sigma -\psi \cdot \frac{G}{B})=\frac{1}{1-{\tilde{S}}^{**}}\cdot {(\Delta \varphi }^{\left(E\right)}-{\psi \cdot \Delta \varphi }^{**}),$$

where ${D^{\left(E\right)}=[C}^{\left(E\right)}{]}^{-1}$ is the inverse of Hook’s matrix, ${\tilde{S}}^{**}$ is the scalar, ${\Delta \varphi }^{**}$ are is the column vector of Green–Lagrange of total true strain, and **G** is a matrix, according to the following formula:

(23) $$G={\frac{2}{3}\cdot \sigma }_{Y}\left({(\varphi }_{e}^{\left(VP\right)},{\dot{\varphi }}_{e}^{\left(VP\right)}\right)\cdot {\dot{E}}_{T}^{\left(VP\right)}\cdot \Delta {\dot{\varphi }}_{e}^{\left(VP\right)}\cdot \tilde{S}.$$

In numerical calculations, the matrix notation of Formula (18) obtained after its discretization by the finite element method is also used:

(24) $$\Delta \sigma =C^{\left(E\right)}\cdot \overset{-}{B}+\left[\Delta q\right]\cdot \tilde{B}\cdot \left\{\Delta q\right\}+\psi \cdot \Delta \sigma^{**}=\left(1-{\tilde{S}}^{**}\right)\cdot \left(\overset{-}{S}+C^{\left(E\right)}\right)\cdot \left[\Delta q\right]\cdot \tilde{B}\cdot \left\{\Delta q\right\}+\psi \cdot \Delta \sigma^{**},$$

where $\overset{-}{B}$ and $\tilde{B}$ are the temporary matrices of the linear and non-linear dependence of the strain increment Δε on the displacement increment of the nodal points Δr, respectively; $\overset{-}{S}=C^{\left(E\right)}\cdot \overset{-}{B}$ is the stress matrix of the discrete system; $\left[\Delta q\right]$ and {Δq} are, respectively, a matrix and a column vector of the displacement increment of the nodal points of the system on a step time.

In the calculation process, it is necessary to accumulate incremental quantities, while the problem of accumulation of the components of the vectors of the displacement increase ${\Delta u}_{i}$, and the components of the true strain increment tensors ${\Delta \varphi }_{ij}$ and the components of the true strain rate increment tensors ${\Delta \dot{\varphi }}_{ij}$ at any time τ is trivial, and it consists in adding their respective components derived in the previous steps.

The accumulation of the effective yield stress increments ${\Delta \sigma }_{Y}$ and the increments of stress tensors ${\Delta T}_{\sigma }$ and their components ${\Delta \sigma }_{ij}$ is not trivial and it requires the use of appropriate algorithms named *“Yield stress accumulation”* and *“Stress accumulation”*.

### 2.5. Experimental Investigations

#### 2.5.1. Samples Tested and Chemical Compositions

**Figure 8:**
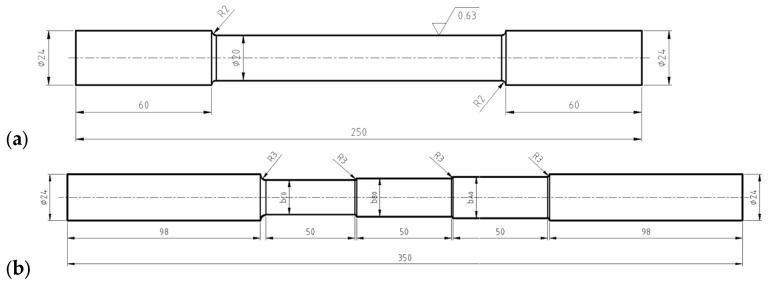
The shape of a cylindrical sample normalized to tension (**a**) and the shape of a three-stage cylindrical sample for determining the constants n and K of the hardening curve (**b**).

**Figure 9:**
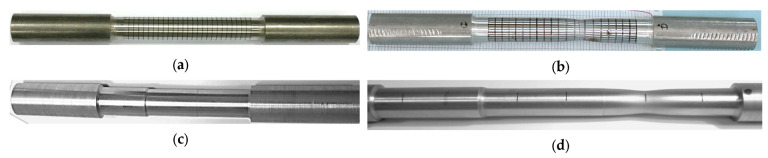
View of samples for testing the mechanical properties of C45 steel: (**a**) one-stage sample, (**b**) one-stage sample after testing, (**c**) three-stage sample, (**d**) three-stage sample after testing.

**Figure 10:**
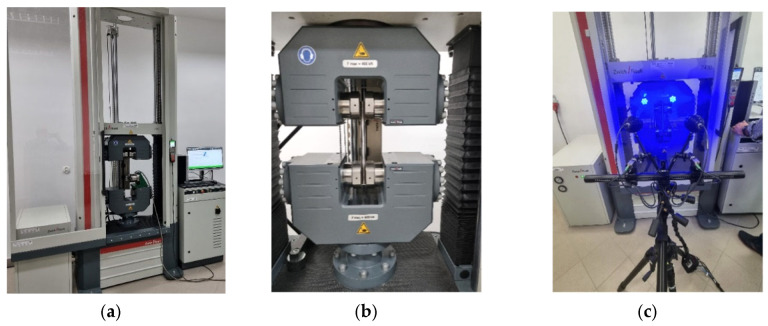
Zwick Roell Z400E testing machine: (**a**) general view; (**b**) view of the traverses with a sample; (**c**) view of the ARAMIS device for optical measurement of the sample deformation.

The tensile strength tests were carried out at an ambient temperature of 18 °C for 5 cylindrical samples made of drawn steel bars in accordance with the PN-EN 10002-1:2004 standard [58] (Figure 8a and Figure 9a) and for 5 three-stage cylindrical samples (Figure 8b and Figure 9b). The material parameters of C45 steel (1.0503) used in numerical calculations were determined in a uniaxial tensile test, on a Zwick Roell Z400E testing machine. The deformation of the samples was measured using the advanced optical measuring system ARAMIS AdjusTable 12M, which uses the technique of digital image correlation, coupled with testing machine and HBM MGCplus data acquisition system. The system allows non-contact measurement of displacements and strains of test samples of any shape and size from 20×15×4 mm to 2500×1800×480 mm. The measurement results are obtained in real time in the full research cycle and the entire field of observation. The data-recording rate is from 25 fr/s to 100 fr/s, the resolution is 12 million pixels (pixel size 3.45 μm). The system is at the Rapid Prototyping Center in Technical University of Koszalin (Figure 10). The influence of strain and strain rate on the yield stress obtained from experiment was compared with the basis of catalog data [59].

The chemical composition was tested in the BATORY Research Laboratory in Chorzów, Poland, by optical emission spectrometry using an optical emission spectrometer type ARL 3460 with spark excitation, manufactured by Thermo Electron in accordance with the PN-H-04045:1997 standard [60]. The tests were carried out at an ambient temperature of 20 °C, measured by a thermo-hygrometer type TFA 30.5031. The chemical compositions of the materials are shown in Table 1 and Table 2.

#### 2.5.2. Effective True Stress-Effective True Strain Curves

In the thread rolling processes, large visco-plastic deformations appear (where: effective true strain $\varphi_{e}^{\left(VP\right)}\leq 2$), is performed with true strain rate of ${\dot{\varepsilon }}_{e}^{\left(VP\right)}\leq 10^{4\;}s^{-1}$[17]. The effect of this strain rate changes the nature of the plastic strain hardening (measured increase of the yield stress—a modulus $E_{T}^{\left(VP\right)}$ and ${\dot{E}}_{T}^{\left(VP\right)}$) and the plastic deformation ability of the material. 

**Figure 11:**
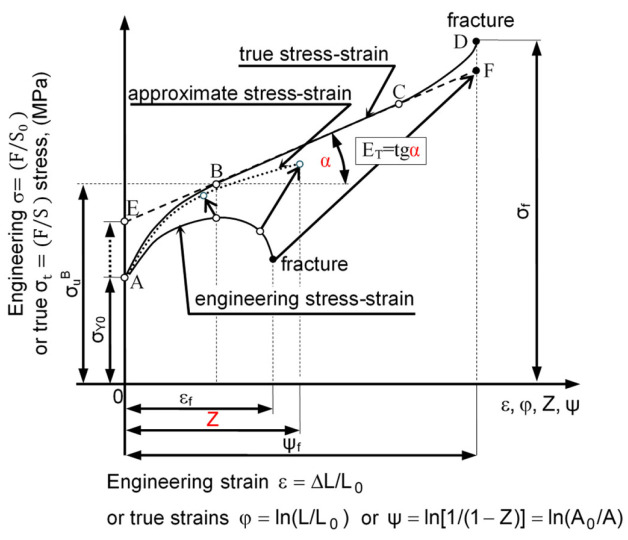
Stress–strain engineering approximate, and true (hardening) curves: ε is the effective engineering plastic strain, φ is the effective true plastic strain, $\sigma_{Y0}$ is the initial yield stress.

In the plastic zone—deformation uniform (Figure 11—section AB), i.e., flow curves for materials insensitive to changing the strain rate, the relationship between stress and strain were described by the following non-linear empirical models: Hollomon’s J.H. [61], Ludwig’s P. [21], and Swift’s H.W. [22].

A fundamental problem is to determine the state of stress and strain of the material beyond the tensile strength at the time of loss of stability of the specimen (i.e., at the time of formation of the neck—Figure 11, point B). The formation of the characteristic throat uniform interferes with uniaxial stress state. In the smallest cross-section of the specimen, radial and circumferential stresses also appear. Thus, there is a three-dimensional stress state. To determine the state of true stress in the neck (Figure 11—section BD), the most known methods used so far were: Bridgman P.W. [23], Davidenkov N.N. and Spiridonova N.E. [24], Siebel E. [25], and Szczepiński W. [26]. The effective true stress $\sigma_{e}$ in the specimen at any time of the tensile test in the section BD and the local stress $\sigma_{z}$ in the z-axis in the smallest cross-section of the specimen, depending on the distance r from the center, are calculated using the Bridgman, Davidenkov–Spiridonova, Siebel, or Szczepiński methods [62]. The use of material parameters calculated with these methods for numerical calculations of physical phenomena occurring in the thread rolling process led to a significant overestimation of force parameters (see Section 2.5.3) and, consequently, to product quality inconsistencies. This can also lead to the destruction of the surface layer of the workpiece. Calculation errors are mainly caused by difficulties in determining the value of the neck radius.

The paper uses a more accurate method of determining stress and strain states in a tensile test at any time and place of cylindrical samples, using the finite element method (FEM) [62,63]. This allows the development of the required material-hardening curve in the form of dependencies between effective true stress and effective true strain, and to determine the required mechanical parameters of the workpiece.

#### 2.5.3. Calculation the Mechanical Material Parameters Using Semi-Empirical Method

**Figure 12:**
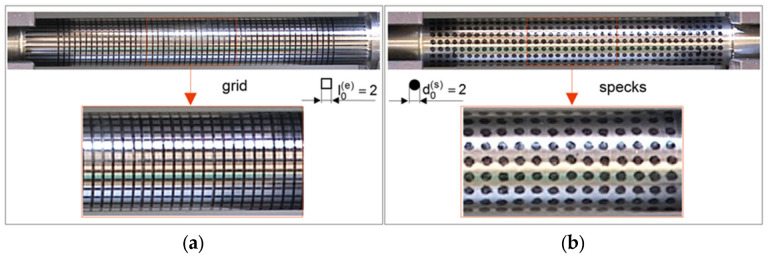
Specimens with grid (**a**) and speck (**b**) coatings [61].

**Figure 13:**
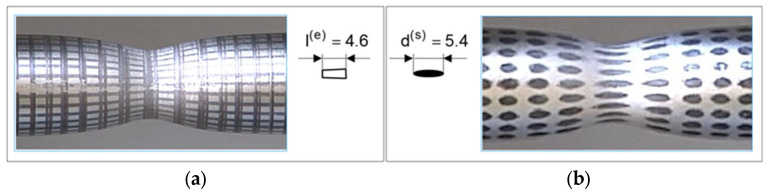
Cylindrical specimens with grid (**a**) and speck (**b**) coatings during tension, with visible necking before the fracture [62].

The application *Numerical tensile test of cylindrical specimen* is developed in APDL in the ANSYS/LS-Dyna system [64]. The application allows for a time analysis of the states of displacements, strains, and stresses in any point of the specimen and at any time t in the duration of the tension test, with the history taken into consideration, for the following data:

- the material-related factors of the specimen: Young’s module, Poisson ratio, initial yield stress, strain hardening modulus, sensitivity to strain rate, strain rate hardening modulus, true failure strain, boundary conditions,
- the geometrical factors of the specimen,
- dynamic or static load.
- the initial state of strains and stresses in the specimen,

The essence of this method is to evaluate the required mechanical parameters of the material over a few stages. The first stage is experimental research on the universal testing machine (Figure 10). A tensile test and measurement of force $F_{D}$ are carried out, as well as elongation ΔL. Then, specimen geometry and displacement (grid) (Figure 12a and Figure 13a) or deformation (specks) of mapped coordination points (Figure 12b and Figure 13b) were measured. Then, the surface area $A=\pi \cdot r^{2}$ of the minimal section of the specimen and the fracture average axial stress $\sigma_{f}$ at rupture also are calculated as the ratio of the tensile force $F_{D}$ to the surface area $F_{D}-$ $\sigma_{f}=F_{D}/A$.

Grid node displacements or speck deformation during the tensile testing are the basis for evaluation of local true strain. The basic mechanical properties of the investigated steel are also determined. Then, in the developed application *Numerical tensile test of cylindrical specimen* in the ANSYS/LS-Dyna ver. 2019R3 system, numerical analysis of the tensile test for the cylindrical specimen is performed. 

**Figure 14:**
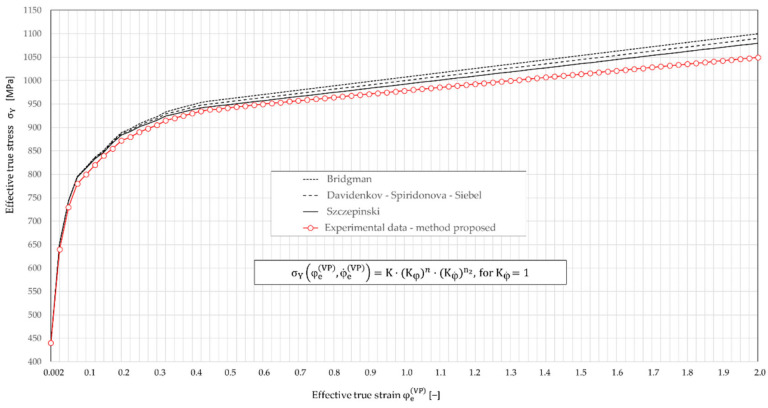
The effective true stress–effective true strain curves developed from the proposed semi-empirical method (Experimental data) and from the Bridgman, Davidenkov–Spiridonova and Siebel, and Szczepiński methods for non-linear strain hardening material models, for C45 steel.

The ability to analyze the tensile test of the specimen step by step not only allows for the determination of stress and strain at anytime and anywhere in the specimen, but also allows for the construction of effective true stress-effective true strain curves $\sigma_{Y}-\varphi_{e}$. In Figure 14, curves $\sigma_{Y}-\varphi_{e}$ for C45 steel non-linear hardening $\left([\partial \sigma_{Y}\left(\varphi_{e}^{\left(VP\right)};{\dot{\varphi }}_{e}^{\left(VP\right)}\right)/\partial \varphi_{e}^{\left(VP\right)}]≅tg\alpha \neq const.\right)$ were developed on the ground of the proposed semi-empirical method are presented, compared and calculated based on the existing methods. 

**Figure 15:**
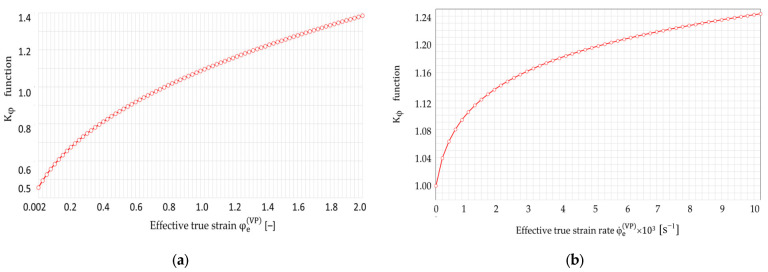
Graphs of one-parameter functions $K_{\varphi }$ (**a**) and $K_{\dot{\varphi }}$ (**b**) in models (8)–(10) for C45 steel, calculated from Formulas (24) and (25), respectively.

Additionally, as a result of the conducted research, the values of constants in one-parameter functions $K_{\varphi }$ in the model (9) and $K_{\dot{\varphi }}$ in the model (11) were determined: $\varphi_{0}=0.049,a=0;b=0;c=0;d=1;e=0;f=0;g=0;h=0.02;n_{2}=0.39;n_{3}=0.6$, which finally have the following forms:

(25) $$K_{\varphi }=0.049+{\left(\varphi_{e}^{\left(VP\right)}\right)}^{0.39},$$

(26) $$K_{\dot{\varphi }}=1+0.02\cdot {\left({\dot{\varphi }}_{e}^{\left(VP\right)}\right)}^{0.6},$$

while the graphs of these functions are shown in Figure 15.

#### 2.5.4. Effective True Stress–Effective True Strain Models

In numerical calculations, in order to calculate the true strain and true stress states at each incremental time step, it is necessary to know the yield stress model of the deformed material. Two material models with non-linear hardening were used in the paper: the first, according to Formulas (9)–(11), and the second, the Cowper–Symonds model, which is normally used in the ANYSYS program [64]. This model takes into account mixed isotropic-kinematic or kinematic plastic hardening and the effect of effective plastic true strain and an effective of plastic true strain rate, according to the Power Relation:

(27) $$\sigma_{Y}\left(\varphi_{e}^{\left(VP\right)},{\dot{\varphi }}_{e}^{\left(VP\right)}\right)={K\cdot (K}_{\varphi }{)}^{n}{\cdot (K}_{\dot{\varphi }}{)}^{n_{1}}={\left[R_{e}+\beta {\cdot E}_{p}(\varphi_{e}^{\left(VP\right)})\cdot \varphi_{e}^{\left(VP\right)}\right]}^{n}\cdot {\left[1+\frac{{\dot{\varphi }}_{e}^{\left(VP\right)}}{C}\right]}^{m},$$

where

- K=1; $K_{\varphi }=R_{e}+\beta {\cdot E}_{p}(\varphi_{e}^{\left(VP\right)})\cdot \varphi_{e}^{\left(VP\right)};K_{\dot{\varphi }}=1+\frac{{\dot{\varphi }}_{e}^{\left(VP\right)}}{C};n_{1}=m,$
- β—hardening parameter, isotropic-kinematic (β=1) or kinematic (β=0) plastic hardening,
- $R_{e}$—initial, static yield stress [MPa],
- ${\dot{\varphi }}_{e}^{\left(VP\right)}$—effective visco-plastic strain rate [s^−1^],
- C—material parameter determining the influence of the true strain rate of plastic deformation [s^−1^],
- $m=\frac{1}{P}$—material constant determining the sensitivity of the material to the plastic true strain rate [‒],
- $\varphi_{e}^{\left(VP\right)}$—effective visco-plastic strain [‒],
- $E_{p}(\varphi_{e}^{\left(VP\right)})$—temporary material parameter dependent on the Young’s modulus of elasticity E and of the temporary plastic true strain-hardening modulus $E_{T}(\varphi_{e}^{\left(VP\right)})$, according to the following formula:

(28) $$E_{p}\left(\varphi_{e}^{\left(VP\right)}\right)=\frac{E_{T}\left(\varphi_{e}^{\left(VP\right)}\right)\cdot E}{E-E_{T}\left(\varphi_{e}^{\left(VP\right)}\right)},$$

where modulus $E_{T}(\varphi_{e}^{\left(VP\right)})$ dependent on the instantaneous effective true strain:

(29) $$E_{T}\left(\varphi_{e}^{\left(VP\right)}\right)=\frac{\partial \sigma_{Y}{(\varphi }_{e}^{\left(VP\right)};{\dot{\varphi }}_{e}^{\left(VP\right)})}{\partial \varphi_{e}^{\left(VP\right)}},\;\mathrm{for}\;{\dot{\varphi }}_{e}^{\left(VP\right)}=0\left[s^{-1}\right].$$

In order to determine the remaining constants, $K,n,andn_{1}$ in model (9), it was first linearized by logarithmization, obtaining the following Regression Equation:

(30) $$\ln[{\hat{\bar{\sigma }}}_{Y}{(\varphi }_{e}^{\left(VP\right)},{\dot{\varphi }}_{e}^{\left(VP\right)})]={lnK+n\cdot lnK}_{\varphi }+n_{1}{\cdot lnK}_{\dot{\varphi }}$$

where $K_{\varphi }$ and $K_{\dot{\varphi }}$ are one-parameter functions according to Formulas (25) and (26), respectively. 

Equation (30), in the system of real variables, can be written in the general form:

(31) $$\hat{\bar{Y}}{{=b}_{o}+b}_{1}\cdot {\bar{x}}_{1}+b_{2}\cdot {\bar{x}}_{2},$$

where 

$\hat{\bar{Y}}=ln[{\overset{\hat{¯}}{\sigma }}_{Y}{(\varphi }_{e}^{\left(VP\right)},{\dot{\varphi }}_{e}^{\left(VP\right)})]$—sought function of yield stress,

$b_{o}=lnK,b_{1}=n,b_{2}=n_{1}—$unknown coefficients of the Regression Equation,

${\bar{x}}_{i}$—input real variables, for i=1 and 2: ${\bar{x}}_{1}={lnK}_{\varphi }$ and ${\bar{x}}_{2}={lnK}_{\dot{\varphi }}$.

Because in the thread rolling process, the effective true strain values vary in the range: $\varphi_{e}^{\left(VP\right)}=0.002÷2.0$ [–] while the effective true strain rate values vary in the range: ${\dot{\varphi }}_{e}^{\left(VP\right)}=0÷10^{3}[s^{-1}]$, the input of real variables ${\bar{x}}_{1}$ and ${\bar{x}}_{2}$ in the model (31) vary within the following ranges: ${\bar{x}}_{1}\in [-0.7765÷0.3221]$ and ${\bar{x}}_{2}\in [0÷1.3863]$.

However, after coding the input variables ${\bar{x}}_{1}$ and ${\bar{x}}_{2}$ according to formula [63]:

(32) $${\overset{˘}{\bar{x}}}_{i}=\frac{2(ln{\bar{x}}_{i}-ln{\bar{x}}_{imax})}{ln{\bar{x}}_{imax}-ln{\bar{x}}_{imin}}+1,i=1,2.$$

Equation (31) takes the form:

(33) $$\hat{\bar{Y}}{{=k}_{o}+k}_{1}\cdot {\overset{\overset{˘}{¯}}{x}}_{1}+k_{2}\cdot {\overset{\overset{˘}{¯}}{x}}_{2},$$

where 

$k_{o},k_{1},\mathrm{and}\;k_{2}$—unknown coefficients of the Regression Equation,

${\overset{\overset{˘}{¯}}{x}}_{i}$—input coded variables, ${\overset{\overset{˘}{¯}}{x}}_{i}\in [-1;+1];and\;i=1,2.$

Then, the experiment was performed according to the two-level plan and with the actual and coded values of the parameters listed in Table 3.

Five-fold repeatability of the tests was used. This task required the following steps to be conducted [65,66]:

1. Determination of variability range of the studied parameters.
2. Choice of the class of the mathematical model.
3. Coding the analyzed parameters.
4. Gathering the experiment results.
5. Elimination of results with gross mistakes.
6. Calculating the inter-row variance and standard deviation.
7. Checking the homogeneity of variance.
8. Calculating the coefficients of regression function.
9. Statistical analysis of the regression function.
10. Examination of the significance level of the correlation coefficient.
11. Checking the adequacy of the mathematical model.
12. Decoding the regression function.

Using matrix calculus, the column vector $\{k\}={\left[k_{0}k_{1}k_{2}\right]}^{T}$ of the unknown coefficients in Equation (32) was calculated from the matrix formula:

(34) $$\left\{k\right\}={\left({\left[\overset{˘}{\bar{X}}\right]}^{T}\left[\overset{˘}{\bar{X}}\right]\right)}^{-1}{\left[\overset{˘}{\bar{X}}\right]}^{T}\left\{\bar{Y}\right\}=\frac{1}{4}{\left[\overset{˘}{\bar{X}}\right]}^{T}\left\{\bar{Y}\right\},$$

where:

- $\left[\overset{˘}{\bar{X}}\right]$—input variable matrix of dimension N×L, for data N = 4 and L = 3,
- ${\left[\overset{˘}{\bar{X}}\right]}^{T}$—transposed matrix $\left[\bar{X}\right],$
- ${\left({\left[\overset{˘}{\bar{X}}\right]}^{T}\left[\overset{˘}{\bar{X}}\right]\right)}^{-1}$—covariance matrix, which reduces to the form: ${\left({\left[\overset{˘}{\bar{X}}\right]}^{T}\left[\overset{˘}{\bar{X}}\right]\right)}^{-1}=\frac{1}{4}I_{3\times 3},$ where $I_{3\times 3}$ is a 3×3 diagonal identity matrix,
- $\left\{\bar{Y}\right\}$—column vector of the average values of the experimental results.

The boundaries of the confidence region for Regression Function (30) were determined from the following formula:

(35) $$\hat{\bar{Y}}\pm t_{kr(\alpha ;f=N-L)}\cdot \frac{S_{R}}{\sqrt{N-L-1}}\cdot \sqrt{\frac{1}{4}{\left\{\overset{˘}{\bar{x}}\right\}}^{T}\{\overset{˘}{\bar{x}}\}},$$

where:

$\hat{\bar{Y}}—$regression equation according to Formula (30),

$t_{kr(\alpha ;f)}$—critical value of Student *t*-test for significance level α = 0.05 and the number of the degrees of freedom f = N − L = 1, 

N—number of measurement points in the experimental design: N = 4,

L—number of unknown coefficients in Regression Equation (30); here: L = 3,

$\left\{\overset{˘}{\bar{x}}\right\}$ and ${\left\{\overset{˘}{\bar{x}}\right\}}^{T}—$column vector of the functions of input variables (test factors in coded form) and its transposition: ${\left\{\overset{˘}{\bar{x}}\right\}}^{T}=[1\;{\overset{˘}{\bar{x}}}_{1}\;{\overset{˘}{\bar{x}}}_{2}]$,

$S_{R}=\sum_{i=1}^{i=N}{\left({\hat{\bar{y}}}_{i}-{\bar{y}}_{i}\right)}^{2}$– residual variance,

$\sqrt{\frac{1}{4}{\left\{\overset{˘}{\bar{x}}\right\}}^{T}\{\overset{˘}{\bar{x}}\}}=\sqrt{\frac{1}{4}\left[1+{\left({\overset{˘}{\bar{x}}}_{1}\right)}^{2}+{\left({\overset{˘}{\bar{x}}}_{2}\right)}^{2}\right]}$

${\hat{\bar{y}}}_{i}$—average values of model outputs for plan points calculated from Equation (30),

${\bar{y}}_{i}—$average values of experimental results.

The test results after statistical processing according to the algorithm presented above were used to develop regression equations in the form (30), and after decoding and delarithmization, the following power form of the function for the yield stress of steel C45 was finally obtained:

(36) $${\hat{\bar{\sigma }}}_{Y}\left(\varphi_{e}^{\left(VP\right)},{\dot{\varphi }}_{e}^{\left(VP\right)}\right)={K\cdot (K}_{\varphi }{)}^{n}{\cdot (K}_{\dot{\varphi }}{)}^{n_{2}}=980\cdot {\left[0.049+{\left(\varphi_{e}^{\left(VP\right)}\right)}^{0.39}\right]}^{0.2322}\cdot {\left[1+0.02\cdot {\left({\dot{\varphi }}_{e}^{\left(VP\right)}\right)}^{0.6}\right]}^{0.1222},\;\left[MPa\right]$$

where $K=980\left[MPa\right];n=0.2322;n_{1}=0.1222$ weredetermined to be the values of the parameters of the Regression Equation.

The model, according to formula (36), does not require the calculation of material hardening modules at each incremental step, as in the case of the Cowper–Symonds model, because the influence of hardening is already included in the values of true strain. The material parameters in the visco-plastic yield stress model (9) were determined on the basis of the results of experimental tests and it was proven that they have a significant impact on the accuracy of the simulation.

The material constant values K and n in the yield stress model (9) were also verified using the Heyer method [67]:

$$n=\frac{ln\left(\frac{b_{C0}}{b_{B0}}\cdot \frac{l_{C0}}{l_{B0}}\cdot \frac{l_{B}}{l_{C}}\right)}{ln\left[\frac{ln\left(\frac{l_{B}}{l_{B0}}\right)}{ln\left(\frac{l_{C}}{l_{C0}}\right)}\right]}=0.2322,$$

$$K=\frac{F_{max}}{b_{C0}\cdot g_{0}\cdot \frac{l_{C0}}{l_{C}}\cdot ln\frac{l_{C}}{l_{C0}}}=980\;[MPa].$$

In an analogous way, as with model (36), the values of the constants in the Cowper–Symonds model (27) were calculated and the following final form was obtained:

(37) $$K_{\varphi }=440+E_{p}(\varphi_{e}^{\left(VP\right)})\cdot \varphi_{e}^{\left(VP\right)},$$

(38) $$K_{\dot{\varphi }}=1+\frac{{\dot{\varphi }}_{e}^{\left(VP\right)}}{200},$$

(39) $${\hat{\bar{\sigma }}}_{Y}\left(\varphi_{e}^{\left(VP\right)},{\dot{\varphi }}_{e}^{\left(VP\right)}\right)={K\cdot (K}_{\varphi }{)}^{n}{\cdot (K}_{\dot{\varphi }}{)}^{n_{1}}=(440+E_{p}\cdot \varphi_{e}^{\left(VP\right)})\cdot [1+\frac{{\dot{\varphi }}_{e}^{\left(VP\right)}}{200}{]}^{0.055},$$

where $K=1;\beta =1;n=1;Re=440\;[MPa];C=200\;[s^{-1}]$; m = 0.055.

**Figure 16:**
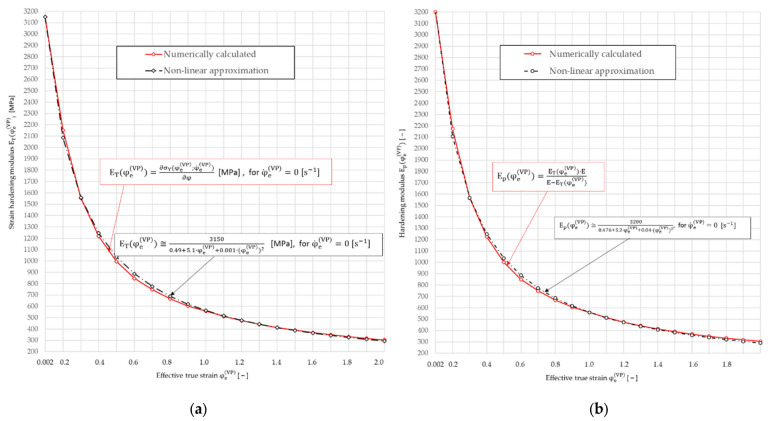
The temporary strain-hardening modulus $E_{T}\left(\varphi_{e}^{\left(\mathrm{VP}\right)}\right)$ [MPa] (**a**) and temporary hardening modulus $E_{p}(\varphi_{e}^{\left(VP\right)})$ [−] (**b**) for C45 steel, obtained numerically according to Formulas (28) and (27), respectively, and from approximation Equations (40) and (41), respectively.

Due to the material non-linearity of the thread rolling process, in the process of step-by-step numerical calculations, the use of the yield stress model (39) first requires the calculation of the temporary strain-hardening modulus $E_{T}(\varphi_{e}^{\left(VP\right)})$ as partial derivative $\partial \sigma_{Y}{(\varphi }_{e}^{\left(VP\right)};{\dot{\varphi }}_{e}^{\left(VP\right)})/\partial \varphi_{e}^{\left(VP\right)}$ from Equation (29), depending on the value of cumulative effective true strains $\varphi_{e}^{\left(VP\right)}$, and then the temporary strain hardening modulus $E_{p}(\varphi_{e}^{\left(VP\right)})$ from formula (28). The calculated values of the temporary hardening modules $E_{T}(\varphi_{e}^{\left(VP\right)})$ and $E_{p}(\varphi_{e}^{\left(VP\right)})$ for C45 steel are presented graphically in Figure 16.

The obtained curves (Figure 14) were approximated by non-linear functions of the following forms:

(40) $$E_{T}(\varphi_{e}^{\left(VP\right)})≅\frac{3150}{0.49+5.1\cdot \varphi_{e}^{\left(VP\right)}+0.001\cdot {(\varphi }_{e}^{\left(VP\right)}{)}^{2}},\;[\mathrm{MPa}],\;\mathrm{for}\;{\dot{\varphi }}_{e}^{\left(VP\right)}=0$$

(41) $$E_{p}(\varphi_{e}^{\left(VP\right)})≅\frac{3200}{0.476+5.2\cdot \varphi_{e}^{\left(VP\right)}+0.04\cdot {(\varphi }_{e}^{\left(VP\right)}{)}^{2}},\;\mathrm{for}\;{\dot{\varphi }}_{e}^{\left(VP\right)}=0.$$

After substituting the dependence (40) into Formula (39), the equation that is convenient to use in numerical calculations was obtained:

(42) $${\hat{\bar{\sigma }}}_{Y}\left(\varphi_{e}^{\left(VP\right)},{\dot{\varphi }}_{e}^{\left(VP\right)}\right)=\left[440+\frac{3200}{0.476+5.2\cdot \varphi_{e}^{\left(VP\right)}+0.04\cdot {(\varphi }_{e}^{\left(VP\right)}{)}^{2}}\cdot \varphi_{e}^{\left(VP\right)}\right]\cdot {\left[1+\frac{{\dot{\varphi }}_{e}^{\left(VP\right)}}{200}\right]}^{0.055}.$$

All the material parameters in the visco-plastic yield stress models (9)–(11) and (27), necessary for numerical calculations, are summarized in Table 4.

**Figure 17:**
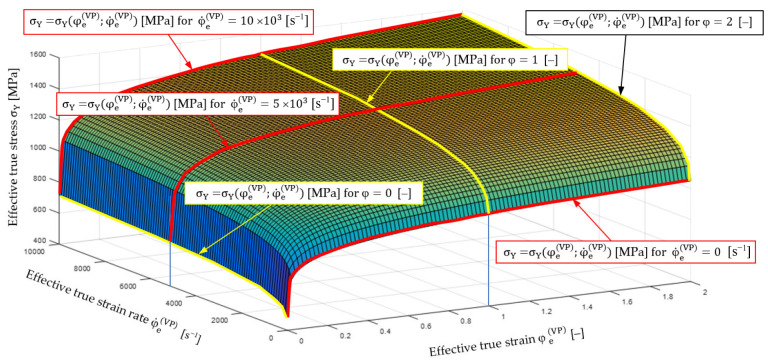
Graphic dependence of yield stresses ${\hat{\bar{\sigma }}}_{Y}\left(\varphi_{e}^{\left(\mathrm{VP}\right)},{\dot{\varphi }}_{e}^{\left(VP\right)}\right)$ for C45 steel in the whole range of variability of true effective strain and true effective strain rate in the thread rolling process determined by the semi-empirical method.

Dependence of yielding stresses ${\hat{\bar{\sigma }}}_{Y}\left(\varphi_{e}^{\left(VP\right)},{\dot{\varphi }}_{e}^{\left(VP\right)}\right)$ for C45 steel in the whole range of variability of true effective strain and true effective strain rate in the thread rolling process determined by the semi-empirical method are graphically presented in Figure 17.

**Figure 18:**
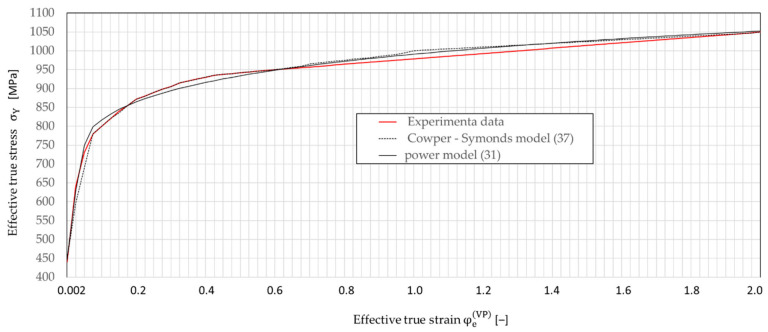
Example graphical relations of plastic stresses ${\hat{\bar{\sigma }}}_{Y}\left(\varphi_{e}^{\left(\mathrm{VP}\right)},{\dot{\varphi }}_{e}^{\left(VP\right)}\right)$ for ${\dot{\varphi }}_{e}^{\left(VP\right)}=0$ for C45 steel obtained on the basis of Equations (36) and (42) and determined by the semi-empirical method.

The ${\hat{\bar{\sigma }}}_{Y}\left(\varphi_{e}^{\left(VP\right)},{\dot{\varphi }}_{e}^{\left(VP\right)}\right)$ relationship obtained by the semi-empirical method (shown in Figure 17) was compared with the relationships based on Equations (36) and (42). It was found that functions (36) and (42) describe this relationship very well, and the correlation coefficient is close to unity (R=0.985). For example, in Figure 18, the graphs obtained on the basis of Equations (36) and (42) for ${\dot{\varphi }}_{e}^{\left(VP\right)}=0$ are presented graphically and compared with the data obtained from the experiment using the semi-empirical method.

#### 2.5.5. Numerical Evaluation of Mechanical Parameters

**Figure 19:**
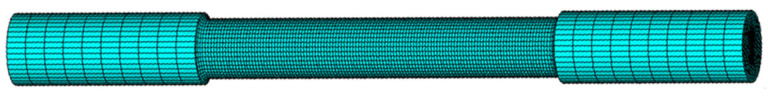
Discretized model of cylindrical specimen.

**Figure 20:**
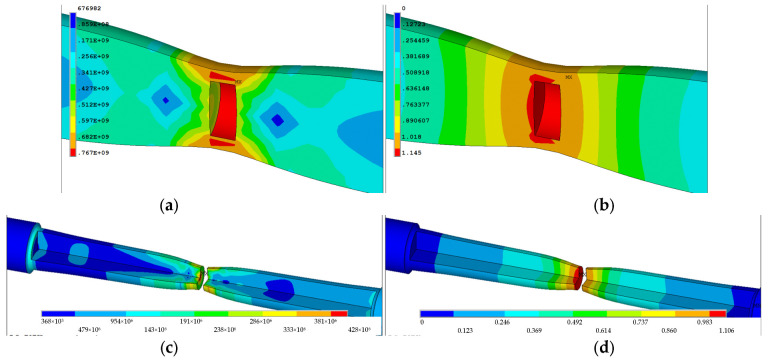
Maps of effective stresses (**a**,**c**) and effective plastic strains (**b**,**d**) before fracture—steps 178 (**a**,**b**) and after fracture—steps 180 (**c**,**d**) in cylindrical specimen from C45 steel.

The verification of the determined material parameters was carried out by numerical analysis of the tensile strength of a standardized cylindrical sample and a comparison of the sample geometry obtained numerically with those measured experimentally. For this purpose, an original application called *“Stretching a cylindrical sample”* in the APDL language in the ANSYS/LS-Dyna program was developed. Figure 19 presents a discrete model of cylindrical specimen applied in the numerical simulation procedure. The model was divided into NE=59,200 finite elements and NN=59,749 nodes with the aid of eight-node elements of the SOLID164 type. As input data for the simulation, material parameters previously determined from the experiment for the power model were introduced. Calculations were performed in 180 steps. In order to control the convergence of the solution, relative values relative to the stress and strain tolerances were established. Figure 20 shows maps of effective stress and effective strain in the specimen at steps 178, 179, and 180. The highest effective stresses occur in the neck before the fracture inside the specimen and are $\sigma_{e}=755$ [MPa] (Figure 20a). However, the plastic strain of the specimen is the largest at the center of the neck and is $\varphi_{e}^{\left(VP\right)}=1.114$ (Figure 20b).

Figure 20 shows selected stages of numerical analysis of the sample stretching process. Figure 20a,b already shows the stage of formation of the so-called “necks”. It is the next stage after the stage of elastic deformation. The material flowed and the neck formed. This was followed by plastic hardening of the material. When the narrowing of the so-called “neck” occurs, there is a stress concentration in this area. The strengthening capacity of the material is no longer high enough to overcome the loss in diameter of the material. After exceeding the permissible deformations, the sample breaks in its smallest cross-section (Figure 20c,d). The greatest stresses occur under the surface of the sample at the fracture site. It is in this area that cracking occurs first, and then it moves outward from the sample.

The ability to analyze the tensile test of the specimen step by step not only allows the determination of stress and strain at anytime and anywhere in the specimen, but also for the construction of curves true stress-true strain ${\sigma_{e}-\varphi }_{e}^{\left(VP\right)}$. In the Figure 17 were presented curve for C45 steel and non-linear hardening developed on the basis of the proposed method. In the calculation, the geometrical dependences on the neck of the specimen were used for step 178 (crack initiation inside specimen), step 179 (crack growth), and step 180 (final fracture) (Figure 20). The obtained values of the effective stresses during sample fracture were compared with the values of stresses calculated on the basis of existing methods (Table 5).

#### 2.5.6. Study of the Mechanics of Plastic Flow during Thread Formation

**Figure 21:**
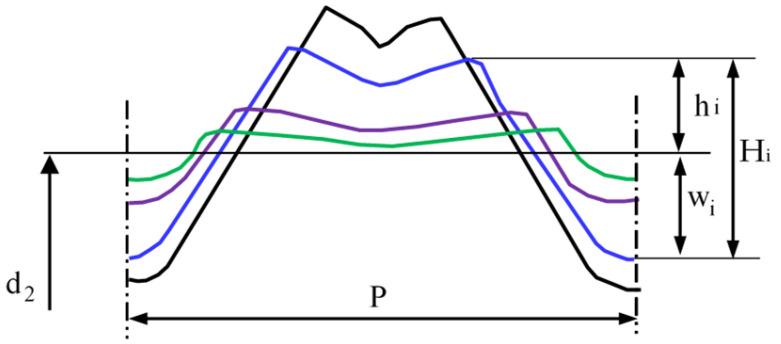
Schema of the course of the thread outline forming: H_i_—i-th height of the thread, w_i_—i-th penetration of the rings, h_i_—i-th hight of the flash, P—thread pitch, d_2_—shaft diameter.

During the thread rolling process, the rolled shaft copies the shape of the profile of the rolls, so some of it moves into the free space between the flanks of the thread profile on the rolls. The degree of space filling (total or partial) depends on many factors, including, among other things, to the outside diameter of the semi-finished product and the rolling interference, and leads to obtaining a thread with a full or incomplete profile. Each rolling ring of the roll occupies a specific position relative to the theoretical outline of the thread to rolled and the surface of the threaded shaft with an outer diameter d_2_ determining the rolling allowance (Figure 21). During rolling, individual rings shape a thread profile suitable for their penetration into the material. At a given moment, this outline is shaped only on a specific part of the circumference, and the loads affect the metal with a frequency depending on the number of rolls and its rotational speed. When the ring of rollers has moved relative to the workpiece, elastic deformation occurs at the beginning, consisting of temporary changes in the distance between atoms in the volume of the crystal lattice and intra-crystalline and inter-crystalline shifts. As a result of the further impact, irreversible displacements of atomic lattice defects occur, causing plastic deformation with the material squeezed out to the sides. To a large extent, the material moves in the radial direction (into the free spaces between the roll rings), gradually increasing the height of the thread. On the other hand, the remaining part of the material is displaced in the axial direction by the action of the feed and the side surfaces of the rolling rings. Until then, it is assumed that the state of displacement and deformation is plane, i.e., it occurs in planes parallel to the axial plane of the object.

**Figure 22:**
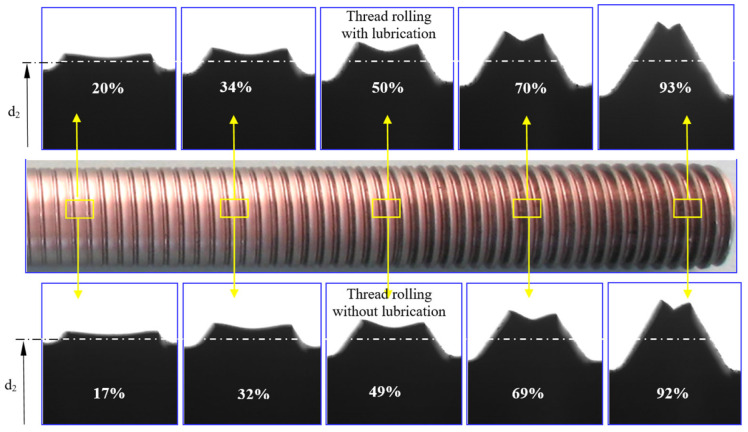
View of a sample with a thread at various stages of the process and axial cross-sections of the thread resulting from the impact of successive rings of the embossing roller on the workpiece, during dry rolling (lower part) and with lubrication (upper part).

When analyzing the shaping of the thread profile during rolling, it can be assumed that the thread profile is symmetrical in various phases of the process. In the initial phase of rolling (Figure 22), a clear difference is noticeable between the displacement of the extreme volumes compared to the middle part of the outline. This is due to the relatively small penetration of the first rolling rings into the workpiece, resulting in an insignificant impact on the central part of the profile. As the next rings penetrated, the entire thread outline was gradually enlarged. The change in the shape and width of the valley on the top results from the increase in the filling of the free space between the roll rings. At the same time, the influence of the lubricant was noticed, which facilitates the movement of the material along the through surfaces of the rolls, causing a slight increase in the height of the thread profile (Figure 22 top) concerning dry rolling, during which the movement of the material is inhibited (Figure 22 bottom). The overlapping of successive outlines obtained after the passage of the first and subsequent rings allows for a schematic representation of the formation of the thread (Figure 21).

The height H_i_ of each of the obtained outlines consists of two quantities:

1. penetration w_i_ of each tip of the roll rolling ring into the shaft, measured from the diameter d_2_ of the semi-finished product,
2. radial outflow h_i_—displacement of the deformed material in the direction opposite to the axis of the thread.

There is a positive correlation between the ring penetration w_i_ and the height of the flash h_i_ for each group of workpieces with similar physical and mechanical properties [9].

Since in the case of rolled threads, an incomplete profile is most often formed, while a full profile is a borderline case, it can assume that the thread is in a plane state of deformation of the material, which was displaced parallel to the axial plane ($Ox_{2}x_{3}$, see Section 3.1.1) of the object. To verify this assumption, experimental investigations were carried out on samples in the form of cylindrical samples made of C45 steel. 

**Figure 23:**
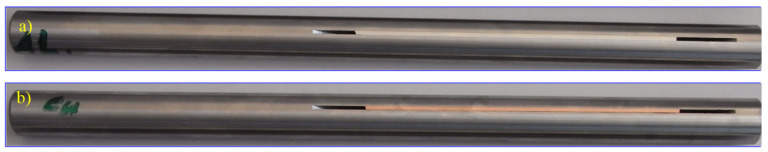
Samples view for the thread rolling with axial inserts of flat steel C45 (**a**) and copper M1E (**b**).

In the semi-finished product, as a grinded shaft, longitudinal axial grooves were created, which were filled by flat steel C45 (Figure 23a) and copper M1E (Figure 23b).

**Figure 24:**
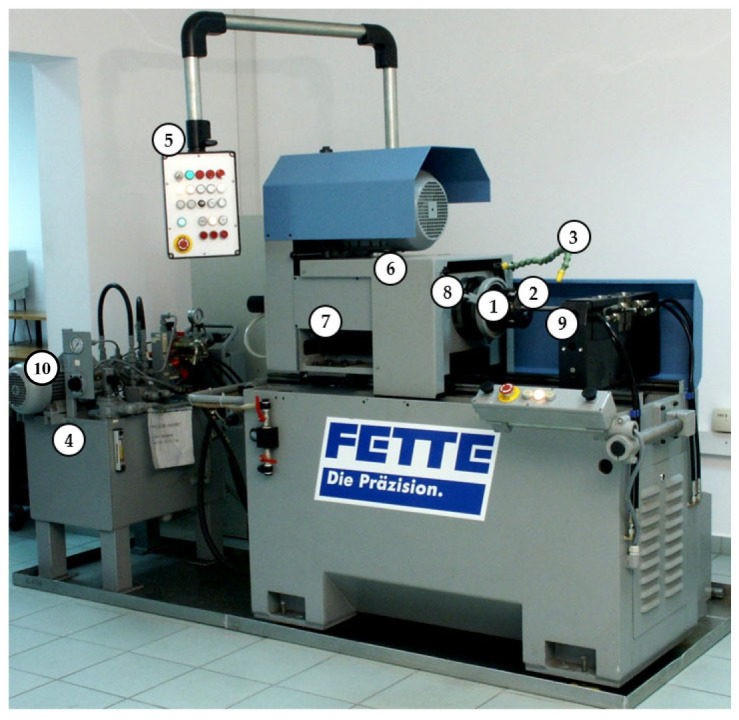
View of the FETTE rolling mill for axial threading: 1—head, 2—workpiece, 3—cooling and lubricating system, 4—hydraulic feeder, 5—control system, 6—fixed headstock, 7—headstock shift control system, 8—control system for closing and opening the head, 9—hydraulic holder, 10—rolling mill motor.

**Figure 25:**
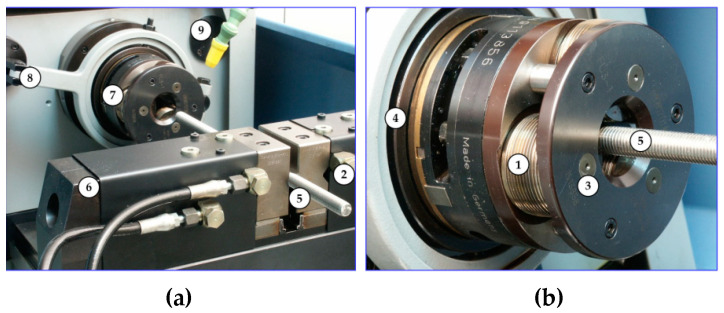
View of the processing system of the FETTE rolling mill for thread-rolling using the axial method (**a**) and view of the rolling head using the axial method (**b**): 1—roller, 2—hydraulic holder, 3—roller axis, 4—thread diameter adjustment mechanism, 5—workpiece, 6—hydraulic vice, 7—three-roller head, 8—head closing and opening system, 9—cooling and lubricating system.

**Figure 26:**
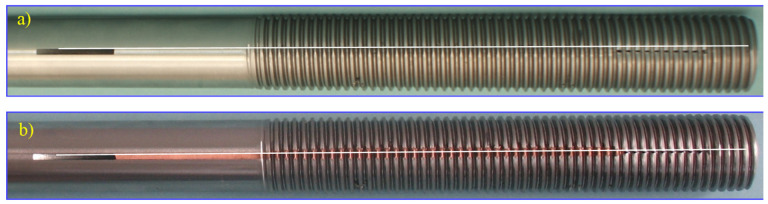
View samples with the thread with flat steel C45 (**a**) and cooper M1E (**b**).

On the bars, the thread was made by the axial method on the stand with an angular head made by FETTE (Figure 24), which contains three rolls (Figure 25). View samples carried out in the thread are shown in Figure 26.

## 3. Results of Experiments

### 3.1. Results of Experimental Investigations

#### 3.1.1. Analysis of the Thread Rolling Process in Real Conditions

**Figure 27:**
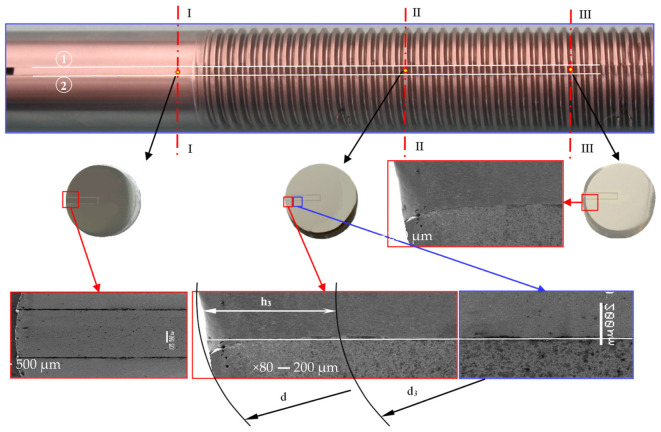
View samples with flat steel insert after rolling and scanning pictures of cross-sectional of the sample at the edge of the groove before rolling (section I-I) and passing through the crest (section II-II) and the root of the thread (section III-III).

After the thread rolling process, the cross-sections of transverse to the axis of the shaft were made in three planes passing respectively through the threadless surface (sections I-I), the top (sections II-II), and the bottom of the thread (sections III-III) (Figure 27).

It has been found that the rectilinear groove planes (1) and (2), which are parallel to the axial plane ($Ox_{2}x_{3},$ Section 3.1.1), also remain parallel after thread rolling. It can be assumed that plane states of displacement and strain occur during thread rolling. Additionally, in cross-sections, the side planes of the grooves remain almost linear. The visible slight curvature of the plane in the upper part of the thread profile results rather from the introduced discontinuity of the material, and not from the spatial state of deformation. The proportion of material at upper part of the profile is only 3.5% of the total thread profile. Thus, with sufficient accuracy for engineering practice, it can be assumed that in the rolled thread material there is a spatial state of stress and plane states of displacement and strain in planes parallel to the axial plane ($Ox_{2}x_{3},$ Section 3.1.1) of the object.

Then, the displacements and deformations of the material during the rolling process can be treated as a series of single identical processes which are characterised by a small contact area (compared to the dimensions of the object and tool) between the active surface of the roll.

Initially, the contact of the tool with the workpiece occurs over small lengths and deformations occur in small volumes. Then, as the process progresses, these zones enlarge, and in the final phase, they cover the entire outline of the thread. Since the length of contact and the volume of the deformed material for a single tooth are relatively small compared to the dimensions of the object and the cylinder, the profile-shaping process can be considered as the contact of a rigid tool with a deformed semi-infinite body.

**Figure 28:**
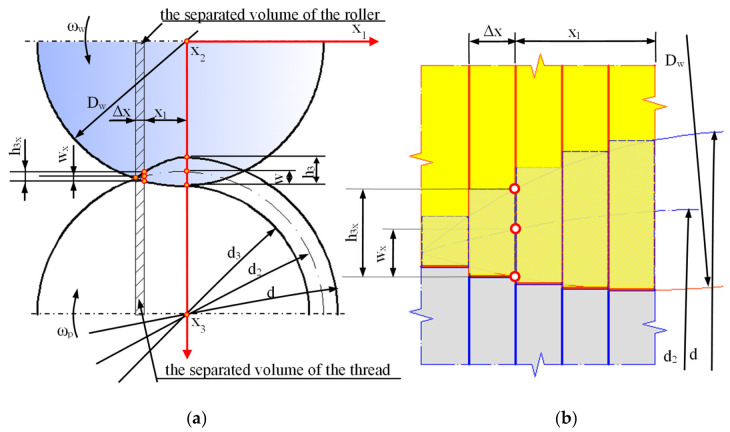
Geometric dependencies in the deformation zone during thread rolling: (**a**) continuous model, (**b**) discrete model.

Under such assumptions, i.e., for a plane strain state, in the Cartesian coordinate system (0xyz) as in Figure 28, the radial strains $\varepsilon_{x}$ and axial strains $\varepsilon_{z}$ are significant, while the circumferential (tangential) strain is negligible $\varepsilon_{y}\approx 0$. This condition, assuming the incompressibility of the object material, leads to the following relationship: $\varepsilon_{z}=-\varepsilon_{x}$.

Any k-th thread profile is treated as a set of elementary volumes (solids) with a very small width $\Delta x_{1}$ $\left(\Delta x_{1}\to 0\right)$. Then, the inclination of the thread profile (resulting from the thread pitch), the outline of its top (with radius d/2), and the variable depression in the workpiece of the cylinder ring at the width $\Delta x_{1}$ can be neglected. Each such volume is separated by two planes, $\pi_{1}$ and $\pi_{2}$, parallel to the axial plane $x_{2}x_{3}$ and spaced from it by the values of $x_{1}$ and $x_{1}+\Delta x_{1}$, respectively (Figure 28). The height $h_{3}$ of the thread profile in this section is:

(43) $$h_{3}\approx 0.5\cdot \left(D_{w}+d_{3}\right)-H,$$

where H is the distance of the axis of the rollers from the axis of the object. 

The length of the base of the isolated solid is equal to the pitch of the thread P.

**Figure 29:**
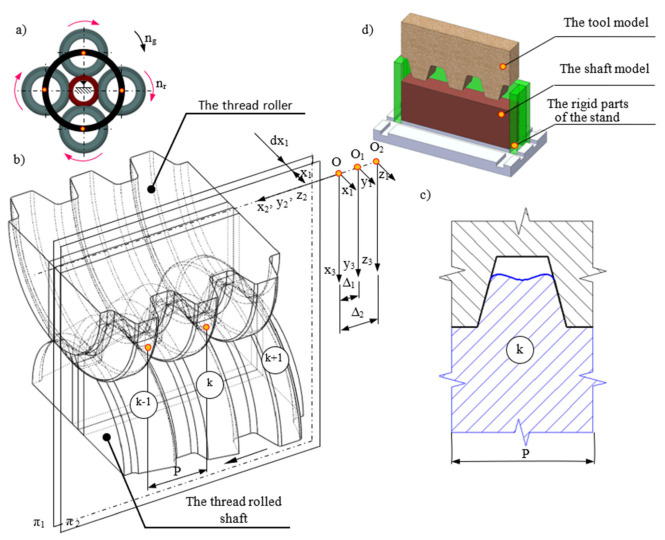
Scheme of thread rolling process using four rolls by axial method (real object) (**a**); scheme of the division of the thread profile and roller rings into solids with a width of dx_1_ (**b**); cross-section of the k-th thread profile and the k-th roller ring with the plane $\pi_{1}$ (**c**); numerical simulation model implementation (**d**).

The planes $\pi_{1}$ and $\pi_{2}$ also limit the part of the active surface of the roller ring with the diameter $D_{w}$ in contact with the considered profile (Figure 29). As in the case of an object, any k-th ring of a cylinder is treated as a set of elementary volumes (solids) with a very small width $\Delta x_{1}$ $\left(\Delta x_{1}\to 0\right)$. Then, the curvature of the top of the ring (with radius $D_{w}/2$) and its variable penetration into the object over the width $\Delta x_{1}$ can be neglected.

The separated part of the cylinder, penetrating into the object to a depth $w_{x}$ equal to:

(44) $$w_{x}=0.5\cdot (D_{w}+d_{2})-w-\sqrt{0.25\cdot D_{w}^{2}-x_{1}^{2}}-\sqrt{0.25\cdot d_{2}^{2}-x_{1}^{2}}$$

where w is the depression of the roller in the axial section, causes the displacement of its material parallel to the plane $x_{2}x_{3}$, shaping the outline of the thread.

#### 3.1.2. Model Tests of the Thread Forming Process

Assuming a plane deformation state and a spatial state of stress, in order to better understand the physical phenomena occurring in the thread rolling process and to determine the mechanism of displacement and strain of the material, model tests were carried out, i.e., on an appropriately enlarged geometric model and with the use of model material.

On the basis made in Section 3.1.1 of the analysis of thread rolling in real conditions, in which there is a spatial state of stress and a flat state of deformation, a stand for model testing of the object (Figure 29d) was developed in which an identical state of stresses and strains of the model material is produced. The purpose of the model tests carried out, i.e., on a properly enlarged geometric model and with the use of model material, was to better understand the physical phenomena occurring in the process of thread rolling and to determine the mechanism of displacement and deformation of the material.

In order to ensure the condition of rheological similarity, yield stresses were determined for the model material—plasticine. This is due to its availability, low price, easy machinability, plasticity (yielding stresses are 100—1000 times lower than the corresponding metal stresses), the possibility of its modification and regeneration, and thus, its repeated use. The basic component of plasticine is calcium carbonate CaCO_3_ and binding agents such as water, mineral, vegetable and animal fats, and other softening and hardening agents and dyes. By selecting the appropriate plasticine composition, it is possible to create various rheological models of bodies (i.e., perfectly plastic, rigid-plastic, strengthening, etc.) as well as multiple versions of the same rheological model with different plastic resistances [27]. The material model was developed based on the static compression test.

**Figure 30:**
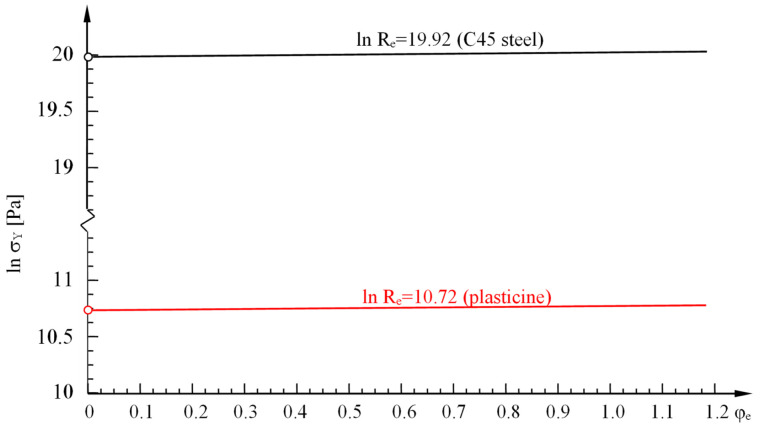
Comparison of the material characteristics of plasticine with the characteristics of C45 steel.

In order to compare the obtained material characteristics of the model material with the characteristics of C45 steel, they were presented in the diagram (Figure 30). Identical inclination angles of the lines in the diagram in the logarithmic coordinate system indicate that the obtained material characteristics for the model material are similar to the material characteristics of C45 steel. Based on the values of the initial yield point determined in strength tests for the model material and steel, which are $R_{e}^{*}=45.63\;[kPa]$ (plasticine), $R_{e}^{}=440\;[MPa]$ (C45 steel), the value of the rheological scale factor is $k_{r}^{}=440,000/45.63=9643$.

**Figure 31:**
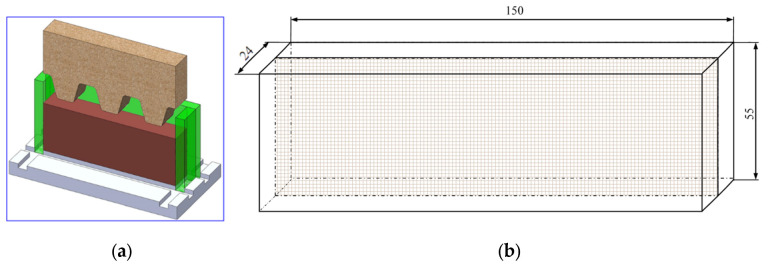
An apparatus for forming the profile of a trapezoidal thread in a flat deformation state (**a**) and a sample for model investigations (**b**).

Flat states of displacement and strain as well as spatial states of stress during model tests of the thread rolling process were obtained by appropriately shaping the tool, punch, and compressed sample (Figure 31a). The two-part sample, shown in Figure 31b, was made of plasticine. On the dividing surface of each half, a grid with shapes, mesh sizes, and location of line families was applied, as in Figure 31b. Intersections of grid lines create material points, from whose displacements the kinematics of the process are determined. Grid lines were applied with a special device.

**Figure 32:**
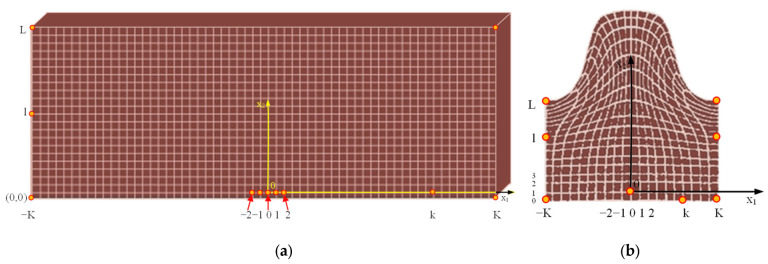
View of the sample with a square mesh (**a**) and view of the sample with a deformed mesh after shaping the trapezoidal outline (**b**).

The coordinates of the nodes were determined in the Cartesian reference system associated with the sample. In the subsequent stages of the process (“step by step”) (Figure 32), i.e., for different values of the punch depth, deformations of the applied mesh were observed and recorded (photographed), and then measured using a WERTH optical microscope with a digital readout with accuracy to 0.001 mm. 

**Figure 33:**
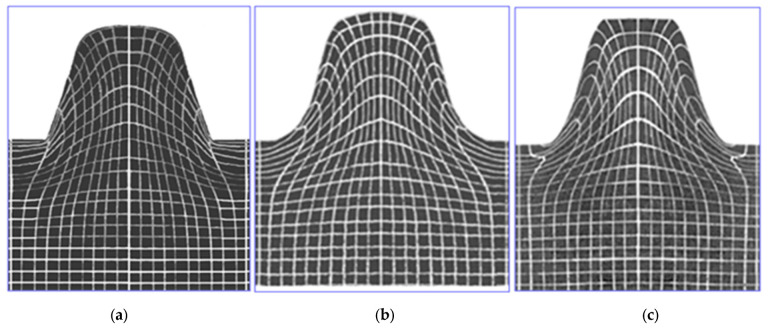
View of the finite element mesh after the trapezoidal-thread-shaping process for different values of the friction coefficient: (**a**)—μ≈0; (**b**)—μ=0.2; (**c**)—μ=0.39.

In model investigations, by changing the surface roughness of the punch, it is also possible to produce different friction conditions, e.g., different values of the friction coefficient (μ≈0÷0.39) in the contact areas of the punch with the model material. In Figure 33, the results of model investigations of the process of shaping the trapezoidal thread profile for various friction coefficients are shown.

Figure 33 shows a significant influence of the friction coefficient on the state of displacements of nodal points, and thus on the state of material deformation. In the absence of friction in the contact area of the tool with the workpiece (Figure 33a), when forming the thread profile, the material is not inhibited by the punch and slides freely along the contact plane. The lack of curvature of the vertical lines of the finite element mesh is visible. An increase in the coefficient of friction results in an increase in the inhibition of material movement. For high values of the friction coefficient (Figure 33b,c), the material is strongly braked in the contact area. There are also areas of material adhesion. There is then greater movement of material in areas further away from the contact zone. This is shown by the curvature of the mesh vertical lines towards the bottom of the thread, mainly in areas close to the contact zone.

**Figure 34:**
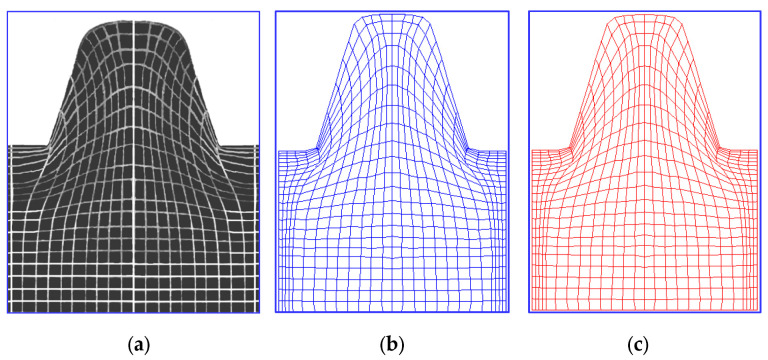
View of exemplary finite element meshes after shaping a trapezoidal thread for the friction coefficient μ≈0: (**a**) after model investigation, (**b**) after numerical simulation according to the first methodology, (**c**) after numerical simulation according to the second methodology.

A numerical application in the ANSYS/LS-Dyna system was also developed, which enables a comprehensive time analysis of deformation states (displacements and strains) occurring in a model object consisting of a rectangular sample and a punch, for spatial states of stress and plane states of displacement and strain. The application allows for the simulation of the process using two methodologies. The first method consists of introducing given boundary conditions in the contact area for displacements determined in model visioplasticity tests (Figure 33). In the second methodology (unknown boundary conditions in the contact area), the incremental theory was used, performing step-by-step calculations. In this case, appropriate modeling of the contact area was performed. The second methodology additionally allows for the determination of the distribution of pressures in the contact area, which was not possible with the first methodology. In this case, the contact area was modeled using CONTA172 and TARGE169 finite elements. The CONTA172 element defines an element that is in contact and slip with the deformed surface defined by the TARGE169 element. The results of the computer simulation were compared with the results of the model investigation, obtaining a very good agreement (Figure 34).

### 3.2. Sensitivity Analysis

System sensitivity analysis is defined as a measure of the change in the system response caused by a change in a selected parameter called the decision variable. In the case of FEM calculations of the TR process, an important step is to determine the sensitivity of the effective maximum strains and stresses at discrete points of the workpiece to: the change in the dimensions of the finite element—shape factor (SF), the number of finite elements (NE), and the shape element function (SEF).

#### 3.2.1. The Influence of the Shape Factor of Finite Elements (SF) 

**Figure 35:**
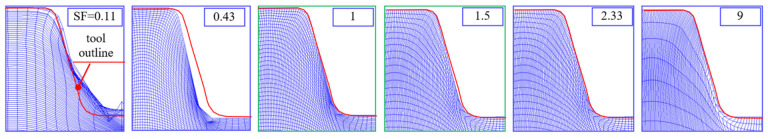
View of the mesh deformation for different values of the shape factor SF.

The shape factor of the finite element SF was defined as the ratio of the height B to the width A of the element: SF=B/A. It is desirable that the aspect ratio be close to unity. Due to the profile of the thread (strong geometric non-linearity, especially at the bottom and top of the thread), discrete models are used, built of finite elements with a large aspect ratio. In order to reduce the number of degrees of freedom of the model, the number of finite elements is minimized by increasing the height of the elements. In order to determine the rational shape of the FE, computer simulations were carried out to determine the impact of SF on the distribution of effective stress and strain and the accuracy of the thread profile mapping. Due to the symmetry of the model, half of the outline was considered. The analysis was carried out using a numerical application developed in the APDL language in the ANSYS/LS-Dyna system. An eight-node element with a non-linear shape function of the PLANE183 type was used to discretize the object. In order to determine the maximum equivalent stresses and strains according to the non-linear Huber–Mises–Hencky (HMH) hypothesis in a trapezoidal thread as a function of the SF, model variants with different division grids were prepared. The following shape factor values were adopted: SF=0.11;0.25;0.43;0.66;1;1.5;2.33;4;and 9. Example results of the simulation of the impact of SF on the accuracy of the tool mapping in the workpiece for the friction coefficient μ≈0 on the contact surface, for the case of a trapezoidal thread, are shown in Figure 35. It was found that the most favorable results of the calculated effective stresses and strains were obtained for SF=1, and therefore, such a value of SF was adopted for further analyses.

#### 3.2.2. The Influence of the Number of Finite Elements (NE)

The next stage of the sensitivity analysis was to determine the impact of the finite element mesh density on the calculation results. In order to determine the mapping of the tool in the workpiece and to determine the maximum effective stresses and strains according to the non-linear HMH hypothesis in the trapezoidal thread as a function of the mesh density, variants of models with different numbers of finite elements were prepared. Models containing finite elements were adopted for the numerical calculations:NE=220;920;3680;8280;22,280;32,880;and 45,600, for SF=1 and μ≈0.

**Figure 36:**
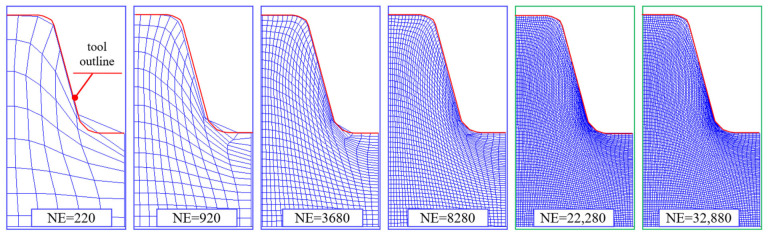
View of the curvature of the finite element mesh (FEM) for a different element number (NE), for the shape factor of finite elements SF = 1.

The impact of the number of finite elements NE on the accuracy of the tool outline mapping is shown in Figure 36. It can be concluded that the use of a minimum NE=22,280 of elements already ensures the correct mapping of the active surface of the tool on the shaft surface. In addition, the increase in the number of finite elements results in a more accurate mapping of the areas of contact between the object material and the tool.

#### 3.2.3. The Influence of the Shape Element Function (SEF)

**Figure 37:**
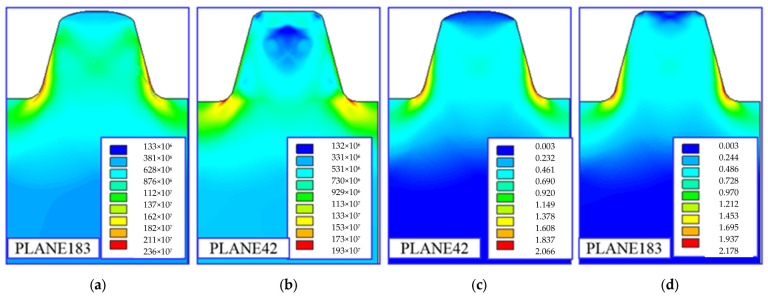
Maps of effective stresses (**a**,**b**) and effective strains (**c**,**d**) for different types of finite elements—different than the shape element function (SEF).

In practice, increasing the accuracy of calculations while reducing the number of finite elements used for discretization can be obtained by increasing the degree of the polynomial, the so-called shape element function (SEF). In order to achieve such an effect, higher-order elements were used in the analyses (elements with eight nodes with a square shape element function—PLANE 183 type) and for comparison, lower-order elements were used (elements with four nodes with a linear shape element function—PLANE42). Example results of calculations of equivalent stresses and strains in a trapezoidal thread are shown in Figure 37.

#### 3.2.4. Effective Discrete Model for Trapezoidal Threads

On the basis of the sensitivity analysis carried out and additionally taking into account the friction conditions in the contact areas of the tool with the workpiece (μ≠0), the following effective model was adopted for further numerical calculations: shape function SF=1; number of finite elements NE=32,280; number of nodes NN=99,433; number of degrees of freedom DOF=198,866 and non-linear of the shape element function—PLANE183 type (Table 6).

### 3.3. Numerical Analysis of the Thread Rolling Proces for a Plane State Deformation 

#### 3.3.1. Computational Model

**Figure 38:**
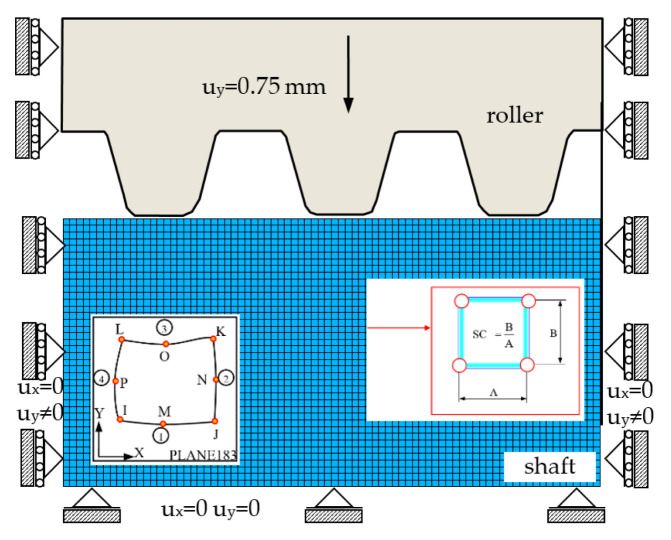
Discrete model for trapezoidal thread rolling.

In the developed application in the ANSYS system, the object was modeled as a plane (2D) state of deformation and a spatial state of stress [10,11,16,18]. Due to the symmetry, half of the profile was considered. The tool was treated as a perfectly rigid body (E→∞) (Figure 38), while the shaft was treated as a non-linear elastic/plastic body with non-linear hardening, which was approximated by the Cowper–Simonds power model (37) (Table 4). The object was discretized with eight-node PLANE183 finite elements with a non-linear shape function. In the contact area, the boundary conditions for displacements are unknown. The application allows for the simulation of the process using the two methodologies presented in Section 3.1.2. In the first method, boundary conditions for the tool and the shaft were selected in accordance with the methodology presented in Section 3.1.1 shown in Figure 29, where the thread rolling depth was selected on the basis of equation (39). The displacement in the y direction was loaded on the tool to a depth equal to half the height for the Tr12×3 thread with trapezoidal outline $w_{y}=u_{y}=0.75$ mm (Figure 38, Table 7). In the second method, the contact of bodies was modelled with the finite elements of the TARGE169 and CONTA172 types. On the bottom of the model, the translational and rotational degrees of freedom were taken off. The calculation was carried out for the material properties from Table 4. Three values of the friction coefficient on the contact surfaces $\mu_{1}=0$, $\mu_{2}=0.2,$ and $\mu_{3}=0.39$ were assumed and the shape coefficient SF=1 (Figure 38, Table 7). The finite elements grid contains NE = 32,880 of finite elements.

#### 3.3.2. Results of Numerical Analysis

**Figure 39:**
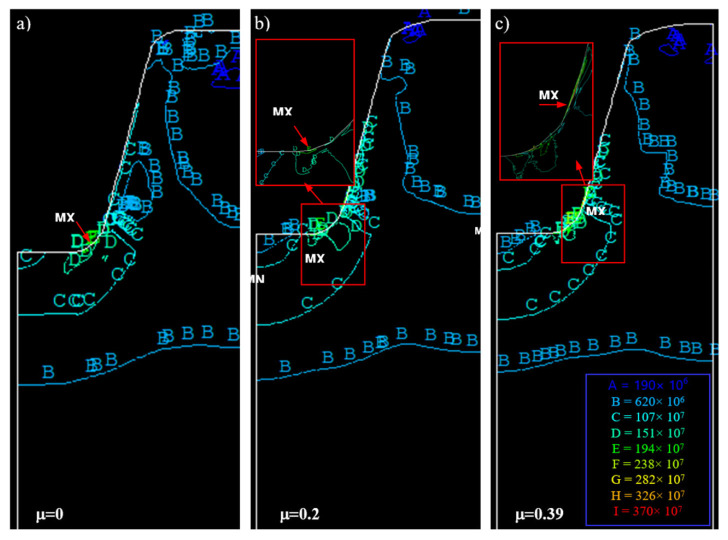
Maps of effective stress for different values of the friction coefficient after a trapezoidal thread rolling, MPa. (**a**) µ = 0; (**b**) µ = 0.2; (**c**) µ = 0.39.

**Figure 40:**
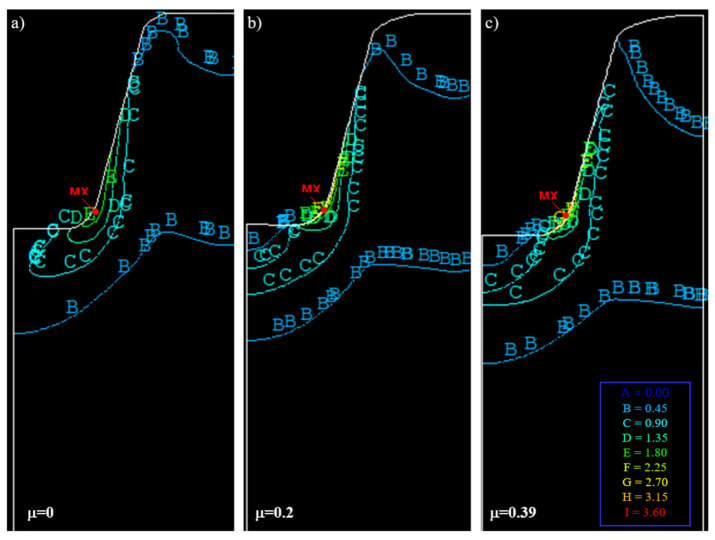
Maps of effective strain for different values of the friction coefficient after a trapezoidal thread rolling. (**a**) µ = 0; (**b**) µ = 0.2; (**c**) µ = 0.39.

Exemplary numerical simulation results for trapezoidal threads are shown in Figure 39 and Figure 40. Analysing the distribution of strain intensity, stress, and deformation of the finite element mesh, a brief influence of lubrication conditions was observed.

The coefficient of friction has a significant impact on the value and distribution of equivalent stresses (Table 8). For μ=0, the highest effective stresses are $\sigma_{e}=2390$ [MPa] and occur at the bottom of the thread on the fillet radius, and based on the thread profile, in the zone adjacent to the contact surface (MX, Figure 39a). With the increase of the friction coefficient, it moves upwards along the contour, taking values from $\sigma_{e}=2410$ [MPa] for μ=0.2 (Figure 39b) to $\sigma_{e}=3610$ [MPa] for μ=0.39 (Figure 39c). For μ>0, a local area of minimum stresses (MN) appears at the bottom of the thread on the axis of symmetry, which assumes a characteristic wedge shape. This minimum, along with the increase of the friction coefficient, moves deeper into the material, covering a larger and larger area.

The coefficient of friction also has a significant impact on the value and distribution of deformations (Table 8). For μ=0, the largest effective deformations are $\varepsilon_{e}=2.058$ and occur (as in the case of stresses) on the fillet radius and the basis of the thread profile (MX, Figure 40a). With the increase of the coefficient of friction, this area moves upwards along the contour, taking values from $\varepsilon_{e}=2.803$ for μ=0.2 (Figure 40b) up to $\varepsilon_{e}=3.514$ for μ=0.39 (Figure 40c). For μ>0, the area of the local minimum (MN) appears at the bottom of the thread on the axis of symmetry, which takes a characteristic wedge shape and is the result of material adhesion. The area of this minimum moves further and further away from the contact surface, going deeper into the material, and covering a larger area as the value of the friction coefficient increases.

## 4. Conclusions

External cold thread rolling with a trapezoidal profile is a complex process in technological terms. This process was analysed as a geometrically and physically non-linear initial and boundary problem with variable stiffness of the system during the rolling process. Boundary conditions are variable in time and space, while in areas of contact between the tool and the workpiece, they are unknown. The measurement of process parameters, such as a displacement zone, temperature, stress, structural change, etc., during the thread rolling process using known techniques of measurement is impossible. Conclusions can only be reached about the properties of the product after the thread rolling.

The currently used methodologies for calculating stress states during the stretching of cylindrical samples result in an overestimation of bursting stresses by about 5%, while the true strains are underestimated by about three times. The applied new semi-empirical methodology allows for accurate determination of the relationship between true stress and true strain and strain rate. This allowed for an increase in the accuracy of the calculations of strain states, strain rates, and stress in the workpiece for the entire range of strain variability and strain rate during the thread rolling process. 

The numerical application of the semi-empirical method proposed allows for more accurate descriptions of the material mechanical properties. It also allows us to determine the moments of cracking (invisible) inside the neck of the sample (Figure 20a,b) and the states of true strain and true stress in the neck.

New constitutive equations were introduced, taking into account the effect of strain and strain rate on material hardening and the non-linear variation of the strain-dependent modulus $E_{T}(\varphi_{e})$ and the second strain-rate-dependent modulus ${\dot{E}}_{T}({\dot{\varphi }}_{e})$ (Figure 17). The proposed method also makes it possible to accurately determine the failure strain and the value of the spring back value after breaking the sample. 

Of the three presented kinematic variants of the rolling process, only the new variant proposed by the Author with a rotating and driven basket ensures the generation of flat states of displacement and strain as well as spatial states of stress in the deformed material.

Model and experimental studies have shown the validity of assuming the presence of a spatial state of stress and plane states of displacement and strain in the rolled thread shaft, occurring in planes parallel to the axial plane. Those made it possible to significantly simplify the numerical model of the process and reduce the calculation time multiple times over.

An application of modern numerical methods and computing systems allows for an analysis of complex physical phenomena occurring in the process under investigation. The application developed in the ANSYS system in the APDL language enables a time analysis of the rolling process with the consideration of the changeability of the roller ring’s profile. 

Based on the course of physical phenomena in the working zone, the technological quality of the thread can be predicted.

Exemplary numerical analyses show that the friction coefficient significantly affects the states of displacements, deformations, and stresses in the surface layer of the screw and is one of the factors determining its technological and functional quality. The best operational quality of the screw was obtained when rolling with abundant lubrication (μ≈0).

The outline of the rollers and the pattern of their penetration into the material are also important. These factors are decisive for the flow mechanics of the object material on which the thread outline and displacement states depend, as well as for the deformations. The results of the numerical simulation can be the basis for the selection of the outline of the working surface of the rolls and the kinematics of the process.

The results of the numerical calculations confirm the possibility of a correct analysis of the material deformation process during thread rolling, both according to method I (set boundary conditions) and method II, without knowing the boundary conditions in the contact area. For the first method, it was necessary to perform model tests using plasticine. However, the limitations of defining the boundary conditions imposed in the computational program are laborious. The disadvantage of this method is also the inability to determine the pressures in the contact area. These inconveniences are eliminated by the second calculation methodology consisting of the introduction of contact finite elements (CONTA and TARGE). An additional benefit of using the second methodology is the development of the pressure distribution in the contact area, which is not possible experimentally.

The results obtained from the numerical simulation can be used to optimise the thread rolling process, reduce design cycles and costs at the beginning of production, reduce overall production costs, and increase product quality. 

The large number of factors affecting the quality of the thread and non-linearity of physical phenomena occurring in the rolling process mean that in order to better understand these factors and determine the mechanism of displacement and deformation of the material, it is advisable to conduct model tests, i.e., on a properly enlarged geometric model and with the use of model material. The developed methodology of model research allows for a better understanding of the physical phenomena occurring in the process of thread rolling. This knowledge is essential for the proper design and control of this complex plastic-forming process.

## Figures and Tables

**Table 1:** Chemical composition of C45 (1.0503) steel.

	Chemical Composition [% Weight]									
	C [%]	Si [%]	Mn [%]	P [%]	S [%]	Cr [%]	Ni [%]	Mo [%]	Cu [%]	Co [%]
Measured	0.44	0.19	0.63	0.007	0.009	0.07	0.06	*–*	*–*	0.01
According to the EN 10083—2 norm	0.42–0.5	0.1–0.4	0.5–0.8	max 0.04	max 0.04	max 0.03	max 0.03	max 0.1	max 0.03	max 0.03

**Table 2:** Chemical composition of Cu-ETP cooper according to the EN 1652/EN 13601/EN 1172 norms.

Chemical Composition [% Weight]								
Ni [%]	Sn [%]	Sb [%]	Pb [%]	Zn [%]	Fe [%]	As [%]	Bi [%]	Cu [%]
max 0.002	max 0.002	max 0.002	max 0.005	max 0.003	max 0.005	max 0.002	max 0.001	min 99.9

**Table 3:** The two-level experiment plan.

Level Plan	Investigated Factors			
	Real Values		Coded Values	
	${\bar{x}}_{1}=\ln K_{\varphi }$	${\bar{x}}_{2}=\ln K_{\dot{\varphi }}$	${\overset{˘}{\bar{x}}}_{1}$	${\overset{˘}{\bar{x}}}_{2}$
1	−0.7765	0	−1	−1
2	0.3221	0	+1	−1
3	−0.7765	1.3863	−1	+1
4	0.3221	1.3863	+1	+1

**Table 4:** C45 steel material parameters used in the numerical simulation.

Equations (8)–(10)	ρ [kg·m^−3^]	E [GPa]	ν [–]	K [MPa]	φ_0_ [–]	φ_f_ [–] (ε_f_ [–])	n [–]	n_1_ [–]	n_2_ [–]
	7800	207	0.28	980	0.049	1.52 (2.88)	0.2322	0.1222	0.39
	n_3_ [–]	a [–]	b [–]	c [–]	d [–]	e [–]	f [–]	g [–]	h [–]
	0.6	0	0	0	1	0	0	0	0.02
Equation (26)	ρ [kg·m^−3^]	E [GPa]	Re [MPa]	ν [–]	C [s^-1^]	P [–]	φ_f_ [–] (ε_f_ [–])	e [–]	f [–]
	7800	207	440	0.28	200	18	1.52 (2.88)	0	0
	n [–]	n_1_ [–]	n_2_ [–]	n_3_ [–]	β [–]	a [–]	b [–]	c [–]	d [–]
	1	m	1	1	1	0	0	$\beta \cdot E_{p}\left(\varphi_{e}^{\left(\mathrm{VP}\right)}\right)$	0
	g [–]	h [–]	m [–]						
	1/C	0	0.055						

where: ρ—mass density of the object material; E—Young’s modulus; ν—Poisson’s ratio; K—hardening parameter; Re—initial yield stress; φ_0_—initial strains; n, n_2_—hardening coefficients depending on the strain; n_1_, n_3_—hardening coefficients depending on the strain rate, m; P—material constants determining the sensitivity of the material to the rate of plastic deformation; C—parameter dependent on the strain rate; a–d coefficients depending on the strain; e–h—coefficients depending on the strain rate; $\varphi_{f}$—true failure strain.

**Table 5:** The fracture stress value for various methods of calculation

Method of Calculation	σ_e_, MPa
Semi-empirical method	1050
Davidenkov N.N.	1100
Spiridonova N.E. and Siebel E.	1090
Szczepiński W.	1080

**Table 6:** An effective discrete model for trapezoidal thread.

Name	Value
SF (shape function)	1
NE (number of finite elements)	32,280
NN (number of nodes)	99,433
DOF (number of degrees of freedom)	198,866
Type of finite element	PLANE183

**Table 7:** Simulation parameters.

Simulation Parameters	Value
Process temperature	20 °C
Type of thread	Tr12×3
Thread-rolling depth	$w_{y}=u_{y}=0.75$ mm
Shaft Material	C45
Roller (tool material)	a perfectly rigid body, E→∞
Type of Finite Elements for shaft and roller	PLANE 183
Type of Finite Elements for contact	TARGE169 and CONTA172
Variety of the friction coefficient	$\mu_{1}=0$, $\mu_{2}=0.2,$ and $\mu_{3}=0.39$
Shape coefficient	SF=1

**Table 8:** The maximum value of obtained numerical results for various values of the friction coefficient.

Friction Coefficient	$\mathrm{Maximum}\;\mathrm{Effective}\;\mathrm{Stress}\;\sigma_{e},\;\mathrm{MPa}$	$\mathrm{Maximum}\;\mathrm{Effective}\;\mathrm{Strain}\;\varepsilon_{e}$
0	2390	2.058
0.2	2410	2.803
0.39	3610	3.514

## Data Availability

Not applicable.

## Abbreviations

DEM	dynamic Element Method
FEM	finite element method
TR	the thread rolling
UL	an updated Lagrange description
